# A Two-Compartment Fermentation System to Quantify Strain-Specific Interactions in Microbial Co-Cultures

**DOI:** 10.3390/bioengineering10010103

**Published:** 2023-01-11

**Authors:** Andreas Ulmer, Stefan Veit, Florian Erdemann, Andreas Freund, Maren Loesch, Attila Teleki, Ahmad A. Zeidan, Ralf Takors

**Affiliations:** 1 Institute of Biochemical Engineering, University of Stuttgart, 70569 Stuttgart, Germany; 2 Systems Biology, R&D Discovery, Chr. Hansen A/S, 2970 Hørsholm, Denmark

**Keywords:** microbial consortia, metabolomics, lactic acid bacteria, *Streptococcus thermophilus*, *Lactobacillus bulgaricus*, bioprocess engineering

## Abstract

To fulfil the growing interest in investigating microbial interactions in co-cultures, a novel two-compartment bioreactor system was developed, characterised, and implemented. The system allowed for the exchange of amino acids and peptides via a polyethersulfone membrane that retained biomass. Further system characterisation revealed a Bodenstein number of 18, which hints at backmixing. Together with other physical settings, the existence of unwanted inner-compartment substrate gradients could be ruled out. Furthermore, the study of Damkoehler numbers indicated that a proper metabolite supply between compartments was enabled. Implementing the two-compartment system (2cs) for growing *Streptococcus thermophilus* and *Lactobacillus delbrueckii* subs. *bulgaricus*, which are microorganisms commonly used in yogurt starter cultures, revealed only a small variance between the one-compartment and two-compartment approaches. The 2cs enabled the quantification of the strain-specific production and consumption rates of amino acids in an interacting *S. thermophilus*–*L. bulgaricus* co-culture. Therefore, comparisons between mono- and co-culture performance could be achieved. Both species produce and release amino acids. Only alanine was produced *de novo* from glucose through potential transaminase activity by *L. bulgaricus* and consumed by *S. thermophilus*. Arginine availability in peptides was limited to *S. thermophilus’* growth, indicating active biosynthesis and dependency on the proteolytic activity of *L. bulgaricus*. The application of the 2cs not only opens the door for the quantification of exchange fluxes between microbes but also enables continuous production modes, for example, for targeted evolution studies.

## 1. Introduction

Interactions between bacteria are common in ecology [1,2] and involve complex mechanisms that are not yet fully understood [3]. Analysing these natural consortia is important because it improves our understanding of fundamental processes, such as bacterial communication [4]; enables community reshaping to gain health and environmental benefits [5]; and opens the door for the application of (synthetic) microbial consortia in biotechnological applications [6]. Consequently, thorough studies have been performed to investigate the application potential of interacting microbes [7,8], leading to the development of natural and synthetic co-cultures for industrial use [9,10,11,12].

Microbial interactions allow for a reduction in individual metabolic burden and are considered beneficial for metabolic productivity. For instance, one strain may provide essential nutritional components to another strain and vice versa [13]. Furthermore, the advantages of cofactor and precursor availability may be created for one microorganism if biosynthetic pathways are shared between two strains [14]. In some cases, increased enzyme activity is also observed [15]. Pande et al. [16] provided experimental evidence for the anticipated benefits and studied the growth performance of a synthetic co-culture that relied on the exchange of essential amino acids. Indeed, the growth of the co-culture outperformed that of the mono-culture in the 24 h experiment. Furthermore, the co-culture was stable despite the presence of non-cooperating cells. Smartly sharing metabolic activity between mutually dependent strains yields improvements in biomass production [17,18,19,20].

Driven by the promising potential of microbial consortia for biotechnological applications, here, whether the toolbox for experimental analyses is already complete or should be complemented with novel devices to elucidate strain interactions inside consortia was evaluated. In particular, the following research trends are anticipated to benefit strongly from knowledge of quantitative exchange fluxes among interacting bacteria, which may be measurable in dedicated devices:

- Computational approaches are being steadily extended to unravel and predict interactions between bacteria [21,22,23]. To improve the simulation results, data from quantitative experiments providing strain-specific information—in particular, strain-specific growth rates, metabolite production, and consumption rates—are essential to validate model qualities, as indicated previously [24,25,26,27,28].
- Synthetic co-cultures should be rationally assembled to achieve the desired targets. This demands knowledge of individual uptake and production rates inside co-cultures for fine-tuning the metabolite exchange rates to prevent bottlenecks in supply and the accumulation of intermediates [29,30].
- Adaptive evolution experiments have been used to improve the performance of strains [31,32] and have been adapted for co-culture systems [33,34,35]. However, to select them for the jointly increased growth of co-cultures, individual adjustments may be necessary, such as the implementation of individual dilution rates to prevent overgrowth and washout scenarios.

Consequently, to meet the demands for strain-specific quantification in co-cultures and to extend co-culture cultivation techniques, several approaches have been developed in recent years:

One approach to obtaining strain-specific rates in co-cultures without disturbing metabolic activities is 13-C metabolic flux analysis [36]. To increase the accuracy of estimated fluxes in co-cultures, elegant methods have already been presented by Gebreselassie et al. [37] based on 13-C-labelled amino acids, and Ghosh et al. [38] used labelled peptides. These methods are restricted to specific metabolic networks or require specific experimental conditions. Interestingly, even higher flux-resolution patterns may be obtained when applying compartment-specific metabolomics [39]. These observations have shed some light on the potential to unravel exchange fluxes between interacting compartments, each hosting different species of a bacterial consortium.

Alternatively, strain-specific information may be obtained by separating the cells of a co-culture after harvesting. If the cell morphology differs significantly, centrifugation may be an appropriate separation approach [40]. However, this is a time-consuming procedure and is, consequently, prone to changes in intracellular states because of ongoing enzymatic activities [41]. The latter may be prevented by the application of proper cell inactivation technologies, which thus far are still missing. Furthermore, related approaches call for the individual development and optimisation of protocols, making them difficult to transfer to other co-cultures.

Other approaches utilise the spatial separation of interacting strains, as reviewed previously [42]. Often, such experimental settings are miniaturised, allowing the verification of multiple synthetic constructs in a parallel manner, thereby restricting sampling volumes. Examples include microfluidic systems [43,44,45] and cell culture plates [46]. Our own studies have indicated that a culture sample of approximately 100 µL is the minimal amount required to quantify the biomass correctly. An additional 100 µL of the supernatant is likely necessary to quantify the metabolites. Hence, the sophisticated and quantitative analysis of interacting cells requires larger reaction volumes than those provided by microfluidic and well-plate approaches. Alternatively, dialysis bioreactors [47] may be applied to cultivate co-cultures in two compartments. However, they incur rather high operational and investment costs and may appear somewhat oversized for studying multiple co-cultures in parallel.

To address these limitations, this study aimed to develop a device for co-culture analysis that provides strain-specific information independent of metabolic activity and phenotype. Systematic strain evaluation was enabled by offering a sufficient sampling volume for extensive analysis, and the device was designed to allow quick assembly.

To this end, a compartmentalised fluid system that allowed the growth of two metabolite-exchanging strains was developed and applied. A strain-specific analysis of growth, production, consumption rates, and intracellular metabolite pools was undertaken. Reflecting the importance of co-cultures in yogurt production, the usability of the system was showcased by investigating the anaerobic interaction between *Streptococcus thermophilus* and *Lactobacillus delbrueckii* subs. *bulgaricus*.

The metabolic activities of the strains are linked to each other: the proteolytic system of *L. bulgaricus* comprises the extracellular proteinase PrtB [48] and intracellular peptidases [49], enabling the strain to gain amino acids from casein, which is likely the reason why the strain loses *de novo* biosynthetic capacities for many amino acids from sugar [50]. The proteinase-negative strain *S. thermophilus* benefits from this relationship as it consumes peptides and amino acids from *L. bulgaricus* [51,52]. The proteolytic system of *S. thermophilus* consists of intracellular and extracellular peptidases [53,54,55], which hydrolyse the peptides supplied by *L. bulgaricus*. Peptide and amino acid transporters have been predicted [53,56] and belong to the ABC binding cassette family [55]. Consequently, amino acids are released from *S. thermophilus*, as measured here [57,58,59]. These lactic acid bacteria are used in industrial processes, such as yogurt and bulk chemical production [60,61], but their interactions are not yet fully understood [54].

## 2. Materials and Methods

### 2.1. Medium Conditions

The synthetic medium (SM) for cultivation (Table S1 in Supplementary Materials) was chosen from a previous study [62]. SM containing lactose is indicated as SM + lactose, and SM containing glucose is indicated as SM + glucose. SM containing casein is denoted as SMcas, and SM containing amino acids is denoted as SMaa.

### 2.2. Strain Cultivation

*L. delbrueckii* subsp. *bulgaricus* ATCC BAA-365 and *S. thermophilus* LMG 18311 were received from Chr. Hansen A/S (Hørsholm, Denmark). Precultures and cultivations were performed in crimp-top serum bottles, as described previously [62]. If predefined dilutions were to be installed in cultivations using crimp-top serum bottles, the related medium was removed and replaced with fresh medium every hour.

For cultivations in two-compartment systems (2cs), precultures were prepared as previously described [62]. Calculated amounts of biomass from one or several precultures were washed twice with 0.9% NaCl solution, and the cell pellets were resuspended in the medium to inoculate each compartment.

### 2.3. Biomass Quantification via the Optical Density Method

Biomass was monitored by optical density (λ = 600 nm) using a photometer (Amersham Bioscience, Ultrospec 10 cell density meter) by applying the biomass/optical density correlation from a previous study [62]. The pH was measured off-line with a pH meter (SevenEasyTM; Mettler Toledo, Columbus, OH, USA) connected to a pH electrode (InLab Semi-Micro; Mettler Toledo, Columbus, OH, USA).

### 2.4. Biomass Quantification via Flow Cytometry

Samples were processed with Tris-HCl (1.3 M) EDTA (0.13 M) pH 8 buffer; stained with 1× SYBR™ Green I nucleic acid gel stain concentrate (Thermo Fisher Scientific, Waltham, MA, USA); analysed with the flow cytometer BD Accuri™ C6 (BD Biosciences-US) equipped with four fluorescence detectors (FL1 533/30 nm, FL2 585/40 nm, FL3 > 670 nm, and FL4 675/25 nm), two scatter detectors, a blue laser (488 nm), and a red laser (640 nm); and correlated to biomass concentration *c_x_* (g_DW_ L^−1^), as described previously [62].

### 2.5. Membrane Unit

A membrane unit with two layers was built from polycarbonate to allow the integration of a polyethersulfone (PES; poly(oxy-1,4-phenylsulphonyl-1,4-phenyl)) membrane (pore size 0.2 µm, 15407-47-MIN; Sartorius, Goettingen, Germany) or a polyamide (PA) membrane (pore size 0.2 µm, 25007-47-N, Sartorius, Goettingen, Germany).

### 2.6. Vessel Bioreactor System

Two vessels (50 mL, 101116; Glasgeraetebau Ochs Laborfachhandel e.K., Bovenden, Germany) were connected to the membrane unit using Teflon tubes (inner diameter, 3 mm) and stirred. Each side was equipped with a mixing pump (Watson-Marlow 101U/R) to circulate the cultivation broth between the vessels and the membrane unit. The vessels and membrane units were maintained at 40 °C. The vessels and tubes were sterilised via autoclaving, and the membrane unit was sterilised via immersion in 70% (*v*/*v*) ethanol for 1 h. The sterile assembled vessel bioreactor system was filled with sterile medium as indicated and warmed up to the cultivation temperature. The biomass was then introduced, and samples were collected using a sterile needle and syringe at the vessel openings.

### 2.7. Tube Bioreactor System

The inlets and outlets of the membrane unit were connected to tubes equipped with a feed and harvest unit. The mixing pump (Watson-Marlow 101U/R) was equipped with a PharMed^®^-tube (Saint-Gobain, Courbevoie, France) with an outer diameter of 4.8 mm, inner diameter of 1.6 mm, and a length of 18 cm, resulting in a volume of 0.4 mL.

An additional connecting tube (Rotilo-silicon tube; Carl Roth GmbH + Co. KG, Karlsruhe, Germany) between the inlet and outlet had an inner diameter of 1.5 mm and a length of 31 cm, which resulted in a volume of 0.5 mL. The feed and harvest tubes had inner diameters of 1 mm.

The particles in the membrane unit were removed using 70% (*v*/*v*) ethanol followed by washing with sterile MilliQ water. The tubes and membranes were sterilised via autoclaving. After connecting the tubes and the membrane unit, the cells were seeded into the system by flushing the cell suspension through the feed until the air was removed. Subsequently, the membrane unit and tubes (without the tubes in the mixing pump) were immersed in water at 40 °C to ensure optimal cultivation conditions.

### 2.8. Continuous Cultivation in the Tube Bioreactor System

Each compartment in the tube bioreactor system was equipped with a feed inlet and an outlet to harvest the cultivation suspension for installing individual dilution rates. Syringe pumps (LA100; Landgraf Laborsysteme, Langenhagen, Germany) were used to ensure feeding to each compartment. To enable accurate harvesting, one outlet was equipped with a drawing syringe pump (LA100; Landgraf Laborsysteme, Langenhagen, Germany), whereas the other outlet allowed the free outflow of the cultivation medium. The harvest was collected for 1 h in an ice-cooled syringe or bottle. A new syringe and bottle were then connected to the harvest for the next sampling. The samples were analysed for biomass via flow cytometry or centrifuged (3 min, 14,000 rpm, 4 °C), and the supernatant was stored at −70 °C for further analysis.

### 2.9. Metabolite Balancing

Equation (1) depicts the mass balance for metabolite *i* which may enter one compartment via diffusion and feed (see Section 3.1.1.), may be produced (or consumed) in the reaction volume *V_R_*, and leaves the compartment via efflux-indexed *production*. Considering equal reaction volumes in each compartment, Equation (2) (process model) was derived as follows:

(1) $$\frac{dm_{i}}{dt}={\dot{m}}_{i,feed}-{\dot{m}}_{i,out}+{\dot{m}}_{i,Diffusion}+{\dot{m}}_{i,production}$$

(2) $$\frac{dc_{i}}{dt}=D\cdot (c_{i,feed}-c_{i})+k_{i}\cdot \left(c_{i,connected\;compartment}-c_{i}\right)+Q_{i}$$

where *m_i_* (kg) denotes the mass of metabolite *I*; *t* (h) denotes the time; *c_i_* (mol L^−1^) denotes the concentration of metabolite *i* in the balanced compartment; *c_i,connected compartment_* (mol L^−1^) denotes the concentration of metabolite *i* in the connected compartment; *D* (h^−1^) denotes the dilution rate; *c_i,feed_* (mol L^−1^) denotes the concentration of metabolite *i* in the feed; *k_i_* (h^−1^) denotes the transport coefficient for diffusion in the membrane unit; and *Q_i_* (mol L^−1^ h^−1^) denotes the metabolic productivities (i.e., the production or consumption of metabolite *i*). As indicated, *k_i_* denotes the trans-membrane transport coefficient resulting from the driving concentration profile between connected compartments.

To exploit the experimental data, Equation (2) was discretised for the time intervals *t*_2_*−t*_1_. The metabolic productivity *Q_i,_*_1_ in compartment 1 was calculated by Equation (3), and the metabolic productivity *Q_i,_*_2_ in compartment 2 was calculated by Equation (4). Indexes *1, 2*, *t*_1_, and *t*_2_ code for the compartments and time points (h), respectively.

(3) $$Q_{i,1}=\frac{\left(c_{i,1,t_{2}}-c_{i,1,t_{1}}\right)}{t_{2}-t_{1}}-D_{1}\cdot c_{i,1,feed}+D_{1}\cdot \frac{\left(c_{i,1,t_{1}}+c_{i,1,t_{2}}\right)}{2}-k_{i}\cdot \left(\frac{\left(c_{i,2,t_{1}}+c_{i,2,t_{2}}\right)}{2}-\frac{\left(c_{i,1,t_{1}}+c_{i,1,t_{2}}\right)}{2}\right)$$

(4) $$Q_{i,2}=\frac{\left(c_{i,2,t_{2}}-c_{i,2,t_{1}}\right)}{t_{2}-t_{1}}-D_{2}\cdot c_{i,2,feed}+D_{2}\cdot \frac{\left(c_{i,2,t_{1}}+c_{i,2,t_{2}}\right)}{2}-k_{i}\cdot \left(\frac{\left(c_{i,1,t_{1}}+c_{i,1,t_{2}}\right)}{2}-\frac{\left(c_{i,2,t_{1}}+c_{i,2,t_{2}}\right)}{2}\right)$$

Hence, the biomass-specific activity *q_i_* (mol L^−1^ h^−1^ g_DW_^−1^) for amino acid *i* was calculated by dividing the metabolic productivity *Q_i_* by the biomass *c_x_*.

If 13-C-labelled amino acids were used, the related production and consumption terms *Q_i_^13^* were estimated as follows:

(5) $$\frac{dc_{i,1}^{13}}{dt}=D_{1}\cdot \left(c_{i,1,feed}^{13}-c_{i,1}^{13}\right)+k_{i}\cdot frac^{13}\cdot \left(c_{i,2}^{total}-c_{i,1}^{total}\right)+Q_{i,1}^{13}$$

where *c^13^* denotes the concentration (mol L^−1^) of the fully 13-C-labelled isotopologues; *c^total^* denotes the total concentration of an amino acid irrelevant to its labelling pattern. For non-labelled amino acids, the sum of m + 0 plus the natural m + 1 background of isotopologues was considered. *frac^13^* (molar 13-C concentration divided by total molar concentration) denotes the fully 13-C-labeled isotopologue fraction of an amino acid pool either in compartment 1 (if *c_i,_*_1_ > *c_i,_*_2_) or compartment 2 (if *c_i,_*_2_ > *c_i,_*_1_).

### 2.10. Reaction Rate Constant of Metabolite Productivity

The consumption rate constant *k_consumption,i_* (h^−1^) for amino acids was derived from the productivity *Q_i_* for each amino acid concentration *c_i_* according to Equation (6).

(6) $$k_{consumption,i}=\frac{Q_{i}}{c_{i}}$$

### 2.11. Determination of Amino Acid Transport Coefficients in the Membrane Unit

To determine the transport coefficient *k_i_*, the feed and harvest flows were disconnected, and compartment 1 was filled with 65 mL of various concentrations of amino acids (pH 6.5), whereas compartment 2 was filled with 65 mL of MilliQ water. A constant mixing pump rate of *r_pump_* = 10 mL × min^−1^ was installed in each compartment. Samples (0.5 mL) were taken from each bioreactor after 0, 5, 10, 15, 20, 25, and 30 min or 0, 5, 15, and 30 min, and amino acid concentrations were quantified using HPLC. The process model of Equation (2) is simplified to Equation (7) for compartment 1, and *k_i_* was identified as the least-square estimate in MATLAB ^®^ (R2020a) (Code S1 in Supplementary Materials).

(7) $$\frac{dc_{i,1}}{dt}=-\frac{dc_{i,2}}{dt}=k_{i}\cdot \left(c_{i,2}-c_{i,1}\right)$$

### 2.12. Determination of the Bodenstein Number

To determine the Bodenstein number (*Bo*) in the membrane unit, bromothymol blue solution with a pH of 7.5 (KK19.3; Carl Roth GmbH & Co. KG, Karlsruhe, Germany) was pumped through each side of the membrane unit at a typical cultivation mixing pump rate of 3.7 mL × min^−1^. Subsequently, 15 µL of 2 M HCl tracer was pulsed into one side of the membrane unit, leading to a colour change.

The experiment was recorded using video. Then, one image of the outlet was decomposed into squares for colour analysis using “imread” from MATLAB ^®^. As the red *r*-values showed maximum variability, related intensities were applied for the mixing studies.

The average residence time (*τ*) and its variance (*σ^2^*) were calculated after the pulse perturbation, as defined by a previous study [63]. To characterise the degree of mixing in the membrane unit, the *Bo* was extracted from *τ* and *σ^2^* (Equation (8)):

(8) $$\frac{\sigma^{2}}{\tau^{2}}=\frac{2}{Bo}+\frac{8}{Bo^{2}}$$

### 2.13. Calculation of the Damkoehler Number

The Damkoehler number (*Da*) is a dimensionless mass balance that was adapted to indicate whether amino acid consumption in a compartment encountered limitations due to low amino acid supply by membrane transport [64]. *Da_I_* (dimensionless) was calculated for each amino acid *I* in a compartment between two subsequent data points (*t*_1_ and *t*_2_) when amino acid consumption and transport in the membrane unit into the compartment were present. A homogeneous distribution of amino acids in the compartment was assumed. *Da* considered amino acid decrease by consumption (*Q_i_*) and washout by dilution (*D*). An increase in amino acid concentration in a compartment was expected from transport across the membrane (see Section 3.6.6.). *Da* depicts the quotient between *Q_i_*, *D* for washout, and the transport rate in the membrane unit for an amino acid *i* as follows:

(9) $$Da_{i,t1-t2}=Da_{consumption}+Da_{dilution}=\frac{-Q_{i,t\;}}{k_{i}\cdot g_{i,t1-t2}}+\frac{D\cdot c_{i,t1-t2}}{k_{i}\cdot g_{i,t1-t2}}$$

Trans-compartment concentration gradients *g_i_* (mol L^−1^) were estimated by considering the arithmetic mean (Δ*c*) of the concentrations between time points (*t*_1_ and *t*_2_) according to Equation (10).

(10) $$g_{i}=\Delta c_{i,connected\;compartment,t_{1}-t2}-\Delta c_{i,t_{1}-t2}$$

The pool turnover rate (*k_membrane unit_* (h^−1^)) of metabolite pools in the membrane unit with the volume *V_membrane unit_* (L) imposed by the circulation of the fermentation broth with a mixing pump adjusted to the rate *r_mixing pump_* (L min^−1^) was calculated as follows:

(11) $$k_{membrane\;unit}=\frac{r_{mixing\;pump}}{V_{membrane\;unit}}$$

### 2.14. Quantification of Extracellular Metabolites

Sugar and lactate concentrations were measured with an isocratic Agilent 1200 series HPLC system (Agilent Technologies, Santa Clara, CA, USA) equipped with a Phenomenex guard carbo-H column (4 × 3.0 mm) and a Rezex ROA organic acid H (8%) column (300 × 7.8 mm, 8 μm; Phenomenex) maintained at 50 °C [62]. Separation was achieved with 5 mM H_2_SO_4_ with a constant flow rate of 0.4 mL min^−1^. Samples were pretreated for the precipitation of abundant phosphate by the addition of 4 M NH_3_ and 1.2 M MgSO_4_ solution followed by incubation with 0.1 M H_2_SO_4_. Absolute concentrations were obtained by standard-based external calibration and normalisation with L-rhamnose as the internal standard.

The amino acid concentrations were determined using an Agilent 1200 series instrument (Agilent Technologies, Santa Clara, CA, USA) [62]. Separation was achieved with an Agilent Zorbax Eclipse Plus C_18_ column (250 by 4.6 mm, 5 µm), which was protected by an Agilent Zorbax Eclipse Plus C_18_ guard column (12.5 by 4.6 mm, 5 µm), according to a previously established method [65]. After automatic pre-column derivatisation with ortho-phthaldialdehyde, fluorometric detection (excitation at 230 nm and emission at 450 nm) was performed. The elution buffer consisted of a polar phase (10 mM Na_2_HPO_4_, 10 mM Na_2_B_4_O_7_, 0.5 mM NaN_3_, and pH 8.2) and a non-polar phase (45% (*v*/*v*) acetonitrile and 45% (*v*/*v*) methanol). The quantification of amino acids was achieved via standard-based external calibration and using 4-aminobutanoic acid as an internal standard at 100 µM to correct for analyte variability.

### 2.15. Quantification of Extracellular and Intracellular Metabolites

For extracellular metabolite quantification via LC-MS/MS, the samples were centrifuged at 20,000× *g* for 3 min at 4 °C, and the supernatant was stored at −70 °C. The samples were then filtered (Centrifugation Units ROTI^®^Spin, MINI-3; Carl Roth GmbH & Co. KG, Karlsruhe, Germany) and mixed (1:1 *v*/*v*) with methanol to precipitate the remaining particles.

Biomass samples for intracellular metabolome analysis via LC-MS/MS were centrifuged at 4500× *g* for 3 min and 4 °C, washed with 0.9% (w/v) sodium chloride solution, centrifuged at 20,000× *g* for 3 min at 4 °C, and the pellet was stored at −70 °C. For metabolite extraction, the pellets were supplemented with 120 µL of 100 µM norvalin to correct for analyte variability, boiled at 95 °C for 4 min, and immediately centrifuged for 20 min at 20,000× *g* and 4 °C. The supernatants were filtered (Centrifugation Units ROTI^®^Spin, MINI-3; Carl Roth GmbH & Co. KG, Karlsruhe, Germany) and stored at −70 °C. The metabolite concentrations in the samples were measured using an Agilent 1200 HPLC system coupled with an Agilent 6410 B triple quadrupole mass spectrometer using an electrospray ion source. Chromatographic separation was achieved according to a previously described method [66]. The metabolite pool concentration was quantified by adding defined amounts of analyte standard to the reaction mixture. Data analysis was performed using MassHunter B.05.00 software (Agilent Technologies), and peaks of isotopologues containing 13-C were checked for interference by comparing samples of cultivation from 12-C and 13-C substrates.

### 2.16. Determination of Amino Acid Composition in Casein

First, 32% HCl (200 µL) was slowly added to casein solution (200 µL), vortexed, and incubated at 100 °C for 24 h. After cooling at 18 °C (1 h), 490 µL of 6.23 mM NaOH was slowly added. The samples were stored at −20 °C until HPLC was used to quantify the amino acid concentrations.

### 2.17. Uncertainty Analysis

The measured data were analysed using Microsoft Excel. The mean and standard deviation were calculated using duplicates and triplicates (STABW.S) using Microsoft Excel.

## 3. Results

### 3.1. Design of the Membrane Unit

#### 3.1.1. Membrane Unit Characteristics

**Figure 1:**
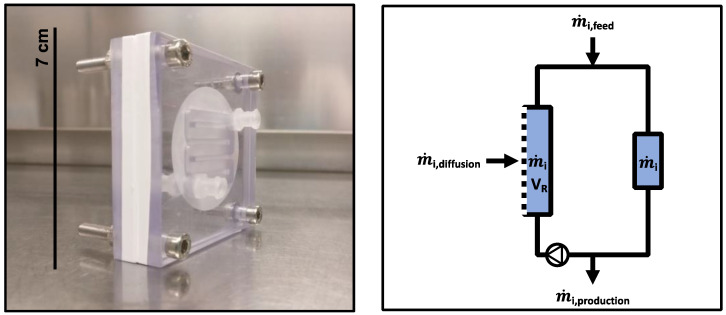
(**left**) Image of the membrane unit. The inlet and outlet of the channel were connected to vessels or tubes to allow the circulation of cells. Two polycarbonate elements were used to clamp a semi-permeable membrane that was aligned and fixed. (**right**) Mass balance of a compartment with inflow (feed), outflow (harvest), and diffusion flows in the membrane unit. The mixing pump allowed the circulation of the cultivation broth within the compartment.

The channels in the membrane unit (see Materials) were located next to each other and were separated by the membrane (Figure 1). This setting enabled the diffusion of metabolites, such as amino acids, but retained the cells. The channel in the membrane unit had a length of approximately 166 mm and volume of approximately 2.7 mL. The inserted membrane area was approximately 6.7 × 10^−4^ m^2^.

#### 3.1.2. Amino Acid Transport in the Membrane Unit

A PES or PA membrane was used to determine the amino acid transport coefficient (*k_i_*) between the two vessels connected by the membrane unit. Three independent experiments were performed. Each experiment contained all of the amino acids. For each experiment, another initial amino acid concentration was set between 150 and 3200 µM (Table S2 in Supplementary Materials). The *k_i_* for amino acid *i* was estimated based on all three experiments (for example, see *k_alanine_* in Figure S1 in Supplementary Materials). The membrane unit equipped with a PES membrane showed a higher mean transport coefficient (*k* = 0.36 ± 0.03 h^−1^) compared to a membrane unit equipped with a PA membrane (*k* = 0.09 ± 0.01 h^−1^) (Figure S2 in Supplementary Materials). Therefore, PES membranes were used in this study. Whether the power input by the mixing pump may bias *k_i_* values by affecting the supply or removal of molecules in the membrane unit was considered. Given a mixing pump rate of *r_pump_* = 10 mL × min^−1^, the average pool turnover rate in the membrane unit was approximately *k_membrane unit_* = 222 h^−1^ on one side of the membrane unit. Considering that the maximum transport coefficients were approximately *k* = 0.4 h^−1^, the fraction of molecules exchanged by diffusion in the membrane unit was *f_diffusion_* = *k*/*k_membrane unit_* = 0.02%. In other words, 99.98% of all the molecules in one compartment of the membrane unit was exchanged via pumping. Reducing *r_pump_* to 3.7 mL × min^−1^ increased *f_diffusion_* to 0.05%, which was still considered to be a low value. Hence, the *k_i_* was barely affected by the pumping rates used in this study.

### 3.2. Design of the 2cs

**Figure 2:**
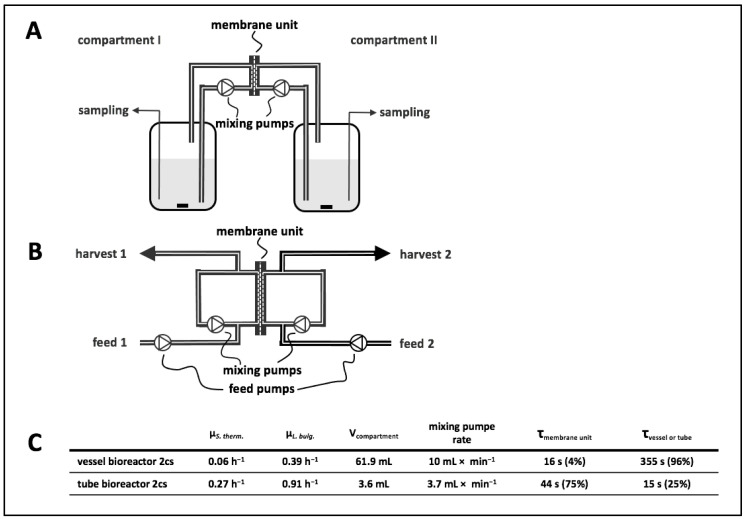
(**A**) Diagram of a vessel bioreactor system. The vessels were connected to the membrane unit, and circulation of medium in each compartment was achieved by mixing pumps. (**B**) Diagram of a tube bioreactor system. The inlets and outlets of the membrane unit were connected by tubes, and circulation of medium in each compartment was achieved by mixing pumps. Additionally, attached tubes for feeds and harvests allowed sampling and continuous cultivation by using feed pumps for each compartment. (**C**) Technical parameters and results of co-cultivations in respective two-compartment systems (2cs) with *Lactobacillus delbrueckii* subs. *bulgaricus* in synthetic medium (SM) containing casein and lactose and *Streptococcus thermophilus* in SM containing lactose. Strains were cultivated in co-culture in the 2cs, enabling exchange of metabolites, and strain-specific growth rates were determined from biomass measurements (Figures S4 and S5 in Supplementary Materials). V, volume.

The presented 2cs was designed to investigate metabolic interactions in a co-culture. This system enabled the characterisation of individual strains by calculating strain-specific rates and quantifying intracellular metabolite pools. As shown in Figure 2, the experimental setup comprised a central membrane unit separating compartments 1 and 2 that may or may not embed an additional vessel section.

### 3.3. Vessel Bioreactor System: Set-Up and Growth Experiment

The vessel bioreactor system comprised two vessels connected by a membrane unit. Each compartment was filled with 61.9 mL of cultivation broth (Figure 2A). To evaluate growth behaviour, compartment 1 was filled with SM + lactose and inoculated with *S. thermophilus*, whereas compartment 2 contained *L. bulgaricus* in SMcas + lactose. The biomass ratio in the 2cs at inoculation was 1:2.75 (g_DW_^LB^:g_DW_^ST^). This experimental setting was chosen to investigate whether the non-proteolytic *S. thermophilus* cultivated in compartment 1 benefited from metabolite exchange with the proteinase-positive *L. bulgaricus* cultivated in compartment 2. Notably, proteinase-negative *S. thermophilus* was not able to grow in SMcas + lactose as a pure culture (Figure S3 in Supplementary Materials). Consequently, the strain crucially relied on *L. bulgaricus*, which released amino acids and peptides from casein that further diffused through the membrane. Considering the geometries and mixing pump rate of 10 mL × min^−1^ in each compartment, the estimated cellular residence time was 355 s in the vessel and 16 s in the membrane unit.

Cultivation studies revealed a growth rate of *µ* = 0.39 h^−1^ for *L. bulgaricus* and *µ* = 0.06 h^−1^ for *S. thermophilus* (Figure S4 in Supplementary Materials). This observation is the first evidence that amino acids and peptides are released from *L. bulgaricus* and that they diffuse into compartments containing *S. thermophilus*. However, the growth of *S. thermophilus* is nutrient-limited.

### 3.4. Tube Bioreactor System

To increase the growth rate of *S. thermophilus*, the vessels were removed from the vessel bioreactor system, leading to a simplified tube bioreactor system design (Figure 2B). Accordingly, the compartment volume reduced from 61.9 to 3.6 mL, increasing the volume fraction in the membrane unit to 74% (instead of 4% in the vessel bioreactor system). By analogy, the membrane-to-compartment ratio improved from 11 m^−1^ in the vessel bioreactor system to 186 m^−1^ in the tube bioreactor system. In other words, the residence time of amino acids and peptides inside the membrane unit increased from 4% to 74% of the total cycling time.

Again, similar experimental conditions were chosen for the first vessel bioreactor system tests; namely, the cultivation of *S. thermophilus* in compartment 1 with SM + lactose and of *L. bulgaricus* in compartment 2 with SMcas + lactose. The mixing pump rate was reduced to 3.7 mL × min^−1^. Dilution rates of *D* = 0.14 h^−1^ were installed in each compartment, resulting in mean residence times of 7.1 h per compartment. The feed medium was equivalent to the medium in the compartments (SM + lactose for feeding into compartment 1 and SMcas + lactose for feeding into compartment 2). The biomass ratio in the 2cs at inoculation was 1:0.7 (g_DW_^LB^:g_DW_^ST^). As expected, the growth of *L. bulgaricus* and *S. thermophiles* was *µ* = 0.91 h^−1^ and *µ* = 0.27 h^−1^, respectively (Figure S5 in Supplementary Materials). For both strains, the growth rates were higher than those in the studies using the vessel bioreactor system.

### 3.5. Comparison between Bacterial Growth in Serum Bottles and in the Tube Bioreactor System

To further characterise the growth of a co-culture in the tube bioreactor system (two-compartments), a crimp-top serum bottle (one-compartment) was additionally inoculated in parallel to the experiment described in Section 3.4. The crimp-top serum bottle contained SMcas + lactose (50 mL) inoculated with the same biomass concentrations of *S. thermophilus* and *L. bulgaricus* and was diluted at the same dilution rate of *D* = 0.14 h^−1^. A defined volume was removed each hour and replaced with new SMcas + lactose medium, imitating the continuous process conditions in the tube bioreactor system described in Section 3.4.

Biomass was determined via flow cytometry at each harvest of the tube bioreactor system and in the crimp-top serum bottle. Then, the cell events of both compartments of the tube bioreactor system were summed up. It was not possible to measure the strain-specific biomass in a one-compartment bottle. As depicted in Figure S6 in Supplementary Materials, the growth of the co-culture in the one-compartment bottle approach was fairly similar to the added-up biomass course in the tube bioreactor system for the first 2 h. Then, exponential growth continued in the tube bioreactor system while the growth rate slowed down in the one-compartment system, finally leading to 3.2 × 10^7^ cell events × mL^−1^ compared to 4.1 × 10^7^ cell events × mL^−1^ in the tube bioreactor system. Apparently, the tube bioreactor system approach was beneficial for the growth of the co-culture.

### 3.6. Determination of Strain-Specific Rates in Co-Culture

To demonstrate the applicability of the tube bioreactor system for identifying exchange rates of metabolites, proteinase-negative *S. thermophilus* and proteinase-positive *L. bulgaricus* were cultivated using medium containing 13-C glucose in the tube bioreactor system. The goal of the experiments was to determine the strain-specific release and consumption of amino acids in the interacting co-culture. Furthermore, experiments were performed to determine whether the released amino acids originated from casein or were synthesised *de novo* from sugar.

#### 3.6.1. Dynamic Cultivation Tests in the Tube Bioreactor System

**Figure 3:**
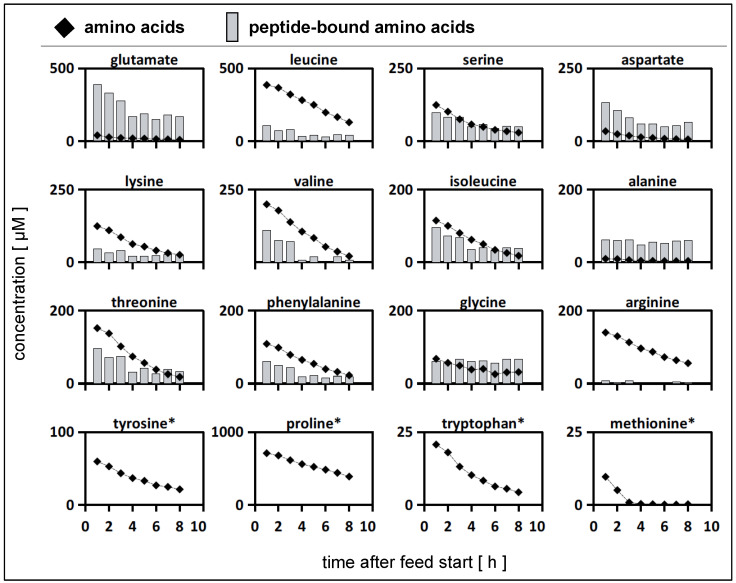
Amino acid profiles in the compartment containing *Streptococcus thermophilus* during co-cultivation with *Lactobacillus delbrueckii* subs. *bulgaricus* in the tube bioreactor system. (**rhomb**) Extracellular amino acid concentrations (µM) in the compartment containing *S. thermophilus* during the continuous mode. (**bars**) Extracellular peptide-bound amino acid concentrations (µM) in the compartment containing *S. thermophilus* during the continuous mode. *S. thermophilus* was cultivated in co-culture with *L. bulgaricus* in the tube bioreactor system containing synthetic medium (SM) with casein and glucose in the *L. bulgaricus* compartment and SM with glucose in the *S. thermophilus* compartment. * Profile data for these peptide-bound amino acids not measured.

*L. bulgaricus* was cultivated in one compartment of the tube bioreactor system containing SMcas + 13-C glucose. In the connected compartment, proteinase-negative *S. thermophilus* was cultivated in SM + 13-C glucose. The experiments were designed such that dynamic growth conditions were set, which were individually adapted to the kinetics of each strain. The biomass ratio in the 2cs at inoculation was 1:4.4 (g_DW_^LB^:g_DW_^ST^). After 2 h of cultivation in the tube bioreactor system, the operational mode switched to continuous fermentation. Pumps feeding the medium with the same composition as the related compartment were started, together with the harvest pump. For the compartment with *S. thermophilus*, a dilution rate of *D* = 0.34 h^−1^ was set to avoid the anticipated overgrowth of the said strain with respect to *L. bulgaricus.* For the latter, a dilution rate of *D* = 0.07 h^−1^ was set to prevent fast washout. After 8 h, that is, 24 h after the start of the experiments, the biomass of each compartment was collected for intracellular metabolite analysis. During the continuous mode period, a mean growth rate of *µ* = 0.05 h^−1^ for *S. thermophilus* and an intermediary maximum of *µ* = 0.1 h^−1^ between 1 and 3 h were observed (Figure S7 in Supplementary Materials). This indicated the growth of *S. thermophilus*, which is only possible in the presence of amino acids or peptides supplied by *L. bulgaricus* (Figure S3 in Supplementary Materials). Therefore, amino acids and peptides must have diffused between the compartments and enriched the medium of *S. thermophilus* (Figure 3). Additionally, the pH dropped in the *S. thermophilus* compartment from 6.5 to 5.5, and lactate production was measured, which revealed the metabolic activity of *S. thermophilus*, *L. bulgaricus*, or both (Figure S8 in Supplementary Materials). Growth and pH were not measured in compartments containing *L. bulgaricus*. Throughout the continuous mode (8 h), *S. thermophilus* and *L. bulgaricus* were replaced 2.7- and 0.6-fold, respectively. In other words, the system did not run under a hydrodynamic steady state. Accordingly, the derived kinetics may serve as operational conditions, demonstrating the feasibility of this approach.

#### 3.6.2. Calculation of Strain-Specific Rates

In co-culture, proteinase-negative *S. thermophilus* consumed peptides and amino acids provided by *L. bulgaricus* to satisfy its nitrogen demand. A previous study using similar strains and experimental conditions [62] demonstrated that co-cultures of *L. bulgaricus* and *S. thermophilus* released and consumed amino acids (as aspartate, arginine, alanine, lysine, isoleucine, and glycine). Consequently, tracking these components may open the door for the identification of strain-specific dynamics and to gain further insight into the interactions of the strains.

**Figure 4:**
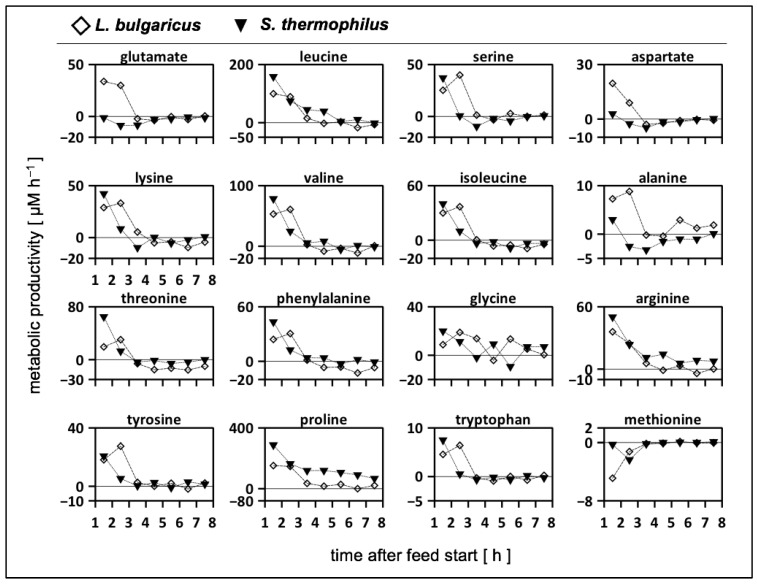
Metabolic productivity of *Lactobacillus delbrueckii* subs. *bulgaricus* (**rhomb**) and *Streptococcus thermophilus* (**triangle**) cultivated in the tube bioreactor system as a co-culture. Positive values indicate the release or production of amino acids; negative values indicate the uptake of amino acids. Strains were cultivated in a tube bioreactor system containing synthetic medium (SM) with casein and glucose in the *L. bulgaricus* compartment and SM with glucose in the *S. thermophilus* compartment. Amino acids were sorted in rows according to the mol-fraction in casein, except tyrosine, proline, tryptophan, and methionine.

The strength of the 2cs is that it allows the calculation of strain-specific amino acid rates by the individual analysis of sample concentrations (Table S3 in Supplementary Materials). As shown in Figure 4, positive values indicate amino acid release regardless of the precursor origin, that is, casein or glucose, whereas negative numbers correlate with amino acid consumption. By trend, both strains released amino acids during the first 3 h before metabolic productivity declined or even before consumption occurred. In particular, *L. bulgaricus* released amino acids (Table S3 in Supplementary Materials) based on its high proteolytic activity. Glutamate, aspartate, and alanine were only produced by *L. bulgaricus* and consumed by *S. thermophilus* during the first 3 h. Another exception was methionine, which was consumed by both strains in the continuous mode.

#### 3.6.3. Biomass-Specific Activity of *S. thermophilus* in Mono- and Co-Cultures

**Figure 5:**
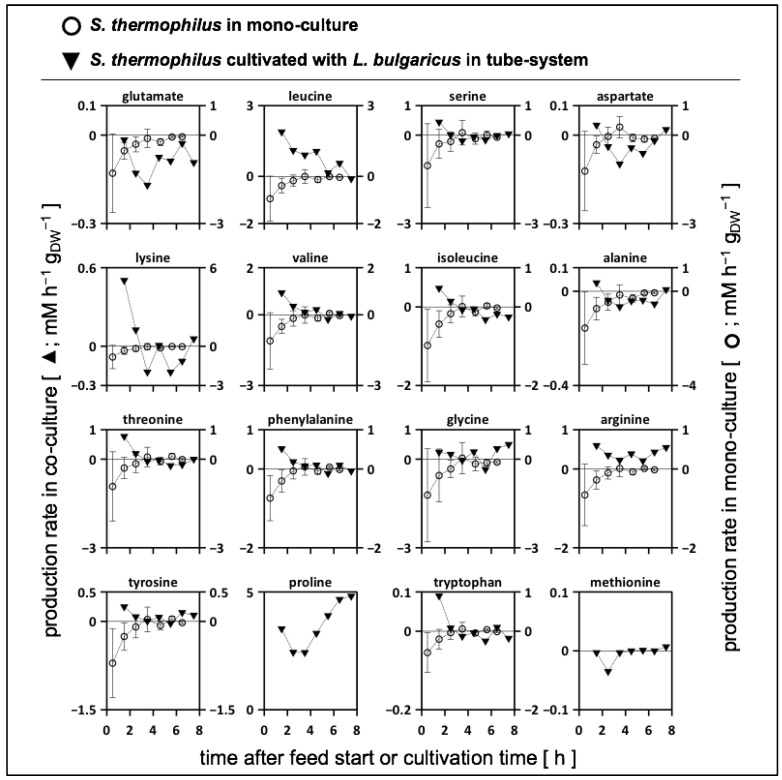
Biomass-specific activity of *Streptococcus thermophilus*. Amino acid production or consumption rates of *S. thermophilus* bridging amino acid productivity in mono-culture and co-culture. (**Filled**) *S. thermophilus* grown in co-culture with *Lactobacillus delbrueckii* subs. *bulgaricus*. Strains were cultivated in a tube bioreactor system containing synthetic medium (SM) with casein and glucose in the *L. bulgaricus* compartment and SM with glucose in the *S. thermophilus* compartment. (**Non-filled**) *S. thermophilus* grown in a crimp-top serum bottle containing SM with amino acids and lactose (modified from [62]). Amino acids were sorted in rows according to mol-fraction in casein, except tyrosine, proline, tryptophan, and methionine.

To gain a deeper understanding of amino acid metabolism in *S. thermophilus*, amino acid productivity has often been studied and modelled [52,67]. However, only strain- and biomass-specific measurements may enable detailed metabolic flux distributions in co-cultures [28], thereby linking mono- and co-culture models [68,69]. Figure 5 compares the amino acid productivity of *S. thermophilus* in a mono-culture grown on SMaa + lactose with the performance when co-cultivated with *L. bulgaricus* in the tube bioreactor system on SMcas + glucose (as shown in Figure 4). Most amino acids were released by *S. thermophilus* in the co-culture, indicating the uptake of peptides as well as intracellular and extracellular peptidase activity [56] compared to the mono-culture condition, where amino acids were almost entirely consumed. Similar to the mono-culture activities, glutamate and aspartate were consumed by *S. thermophilus* in the co-culture. This is remarkable, as peptide-bound glutamate and aspartate are available (Figure 3) but are not preferred. Apparently, *S. thermophilus* prefers consumption rather than replenishing its demand via the hydrolysis of peptides or interconversion through transaminases [70,71]. Methionine was consumed by *S. thermophilus* in the co-culture, but uptake was limited by low methionine concentrations (Figure 3), which might indicate an insufficient supply [67].

#### 3.6.4. Analysis of Extracellular 13-C Alanine Enrichment 

**Figure 6:**
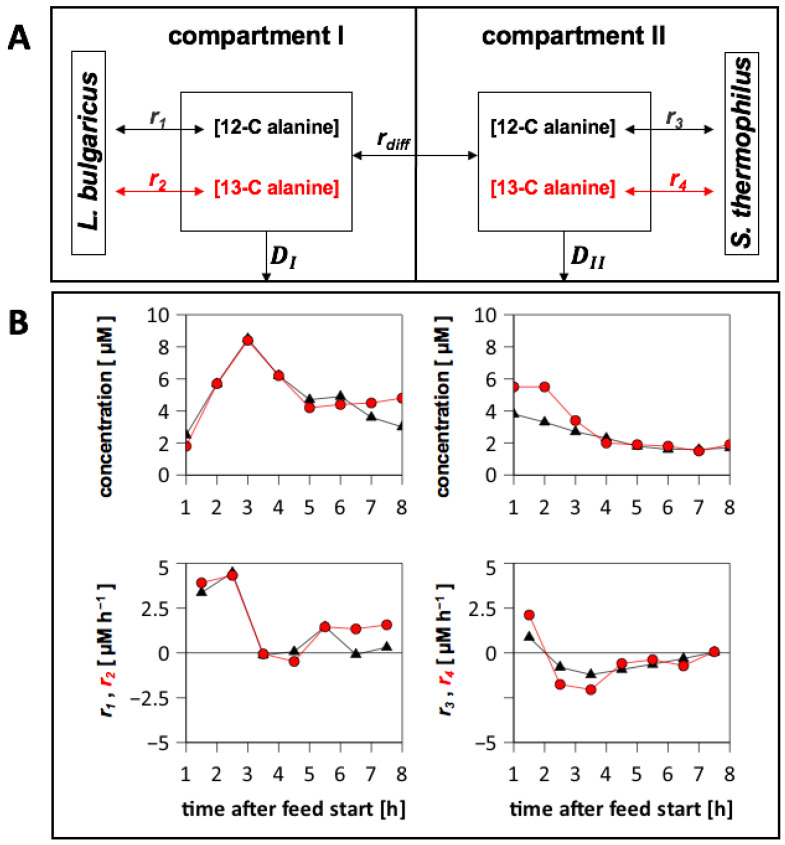
Alanine production and consumption of *Streptococcus thermophilus* and *Lactobacillus delbrueckii* subs. *bulgaricus* cultivated in the tube bioreactor system. (**A**) Illustration of alanine pools in the tube bioreactor system. *r*_1_ and *r*_3_ are the production and consumption rates of non-labelled alanine; *r*_2_ and *r*_4_ are the production and consumption rates of 13-C alanine; *r_diff_* is the diffusion rate of alanine in the membrane unit according to concentration differences; and *D* is the dilution rate in compartment 1 or compartment 2. (**B**) Compartment 1 was filled with *L. bulgaricus* and synthetic medium (SM) with casein and 13-C glucose. Compartment 2 was filled with *S. thermophilus* and SM with 13-C glucose. Concentrations of non-labelled (**triangle**) and 13-C alanine (**circle**) were measured via LC-MS. Strain-specific rates were calculated by balancing each compartment. Positive rates: production; negative rates: consumption.

Concentrations of extracellular amino acid isotopologues were measured to determine the origin of the amino acids. Low fractions of labelled aspartate, tyrosine, and threonine were detected (< 1%). Only the alanine pool (mol L^−1^) was enriched with up to 50% 13-C alanine (Figure 6), which was mirrored by intracellular labelling patterns in both strains (Figure S9 in Supplementary Materials). This observation highlighted the relevance of *de novo* alanine biosynthesis from (labelled) sugars. The strain-specific production and consumption rates for 13-C alanine were calculated (Equation (2)) using the process model (Figure 6A). Balancing revealed that alanine was produced *de novo* by *L. bulgaricus* at a maximum rate of 5 µM × h^−1^, whereas *S. thermophilus* mainly consumed the amino acids (Figure 6B).

#### 3.6.5. Alanine Exchange between the Compartments

The diffusion flux of 13-C alanine across the membrane was calculated. Figure S10 in Supplementary Materials shows a 13-C alanine flux from the compartment containing *L. bulgaricus* to the compartment containing *S. thermophilus* between 2 and 7 h. This indicated that *L. bulgaricus* provided *de novo*-produced alanine to *S. thermophilus* because *S. thermophilus* consumed alanine within this time range (Figure 6).

#### 3.6.6. Calculation of Damkoehler Numbers

**Figure 7:**
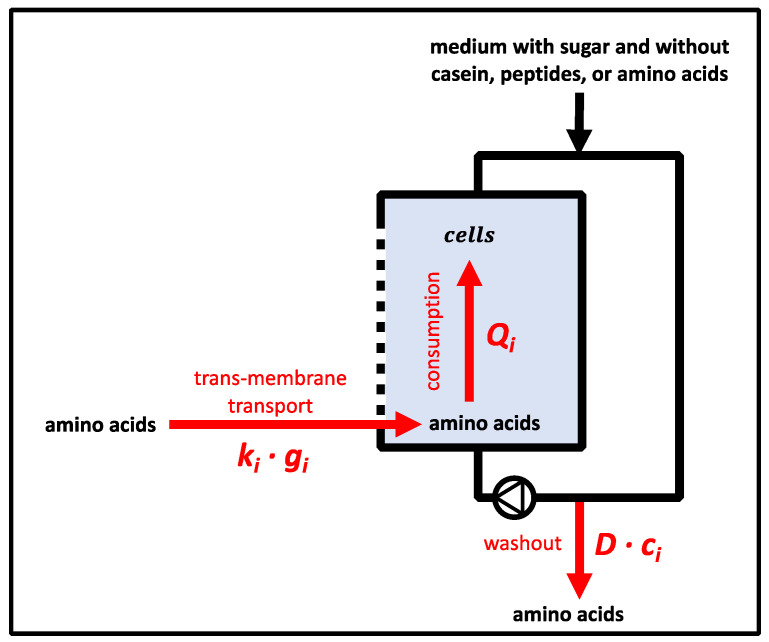
Illustration of terms to estimate the Damkoehler number (*Da*) during the continuous mode. Trans-membrane transport provided amino acids; *Streptococcus thermophilus* or *Lactobacillus delbrueckii* subs. *bulgaricus* consumed amino acids; and the continuous mode provoked amino acid washout. The initial concentration for some amino acids was above zero at the start of the continuous mode.

To further investigate the metabolite dynamics in the continuous experiments, Damkoehler numbers were calculated for each amino acid (Figure 7). In essence, the terms for amino acid consumption and washout were compared with trans-membrane amino acid transport rates, leading to *Da_consumption_* and *Da_dilution_*, respectively (Table 1). Accordingly, *Da* < 1 indicated a faster amino acid supply than depletion, and this was the opposite for *Da* > 1, whereas *Da* = 1 represented an equilibrium between depletion and supply. The calculation of the *Da* terms *Da_consumption_* and *Da_dilution_* (Equation (9)) illustrated their individual importance for the total *Da* term.

**Figure 8:**
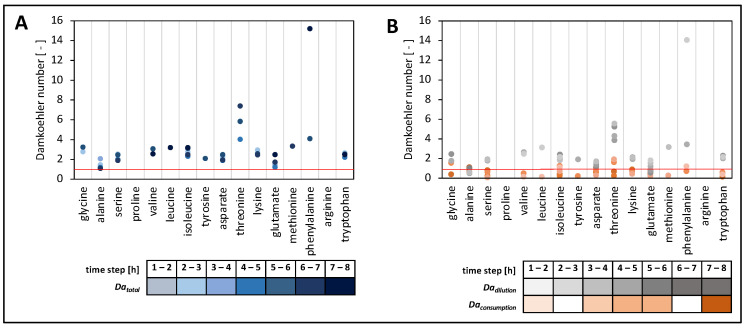
Damkoehler numbers (*Da_I_*) of individual amino acids. (**A**) *Da_total_* = *Da_dilution_* + *Da_consumption_* in the compartment containing *Streptococcus thermophilus*. (**B**) *Da_dilution_* + *Da_consumption_* separated in the compartment containing *S. thermophilus*. Strains were cultivated in the tube bioreactor system, and *Da* was calculated for each hour of continuous cultivation. *Da* numbers were only calculated if amino acid uptake was present within 1 h. The red line indicates *Da* = 1.

The analysis of *Da*_total_ time courses for the compartment containing *S. thermophilus* revealed that *Da_total_* data were > 1 (Figure 8A) for all amino acids, irrespective of the time interval. By trend, the highest *Da_total_* values were observed after 5 h, with alanine being the only exception. Consequently, most amino acids showed greater concentration decreases than their supply from the compartment containing *L. bulgaricus.* This scenario was only enabled by the already high concentrations of these amino acids within the compartments at the start of the continuous experiment (Figure 3). In the case of alanine, sugar-derived biosynthesis became more important as the experiment lasted longer. Figure 8B discloses the individual contributions of *Da_dilution_* and *Da_consumption_* for the calculation of the total *Da* number *Da_total_* showcasing the compartment of *S. thermophilus*. *Da_dilution_* was larger than *Da_consumption_*, outlining that the decrease in amino acid concentrations was predominately caused by the washout of amino acids (*D* = 0.34 h^−1^) and not by their consumption (*k_consumption_* = 0.15 ± 0.16 h^−1^) (Figure 7).

## 4. Discussion

### 4.1. Process Characterisation

The fluid behaviour in the membrane unit can be described by *Bo* = 18 (Figure S11 in Supplementary Materials). This indicated that axial molecular diffusion and additional backmixing effects were present [63]. Given that *Bo* represents the ratio between convective flow and axial backmixing (dispersion), one may estimate that a non-optimum plug-flow pattern exists inside the channels with approximately 5% backmixing. Backmixing increased the average residence time of elements inside the membrane unit. However, 5% is far too low to create substrate gradients inside the compartment, as consumption rates are much lower than the sum of trans-membrane transport (Table 1).

To investigate whether the diffusion process of metabolites in the membrane unit might result in limitations, such as the supply of amino acids from *L. bulgaricus* to *S. thermophilus*, Damkoehler numbers were estimated according to Equation (9). As almost all *Da_total_* values were > 1, indicating stronger amino acid withdrawal than supply, cellular growth predominately relied on the amino acids that were released at the beginning of the continuous experiment or those that were already present before the start (Figure 3). However, the key readouts regarding amino acid dependencies could be deduced. Nevertheless, future experimental settings may reduce the dilution rate *D* as the key parameter for washout, which would significantly reduce the available amino acid amount per compartment (Figure 7).

### 4.2. Difference between Cultivation in the Serum Bottle and in the Tube Bioreactor System

The growth of the co-culture in the serum bottle and in the tube bioreactor system was compared to study the potential impacts of hampered cell-to-cell interactions. Metabolic interactions could be delayed because of diffusion-limited metabolite exchange, and missing cell-to-cell contact may create secondary responses [72]. Interestingly, 33% more cell events, that is, the proxy for cell growth, were found in the tube bioreactor system, which might have been the result of delayed acidification (Figure S6 in Supplementary Materials). Like amino acids, lactate needs to cross the membrane unit via diffusion, which decelerates acidification dynamics in the connected compartment while maintaining beneficial pH conditions for growth.

### 4.3. Strain-Specific Amino Acid Release and Consumption in the Tube Bioreactor System

Both strains released and consumed amino acids when cultivated in a tube bioreactor system in continuous mode (Figure 4). During the first 3 h, both strains mainly released amino acids. Subsequently, amino acids were released and consumed. Only methionine was entirely consumed during the continuous mode. These findings quantified, for the first time, to our knowledge, the amino acid production and consumption rates in an interacting co-culture of *L. bulgaricus* and *S. thermophilus* and highlighted their dynamics. Consequently, the amino acid transport demonstrated for both strains and their impact on proton gradient and energy metabolism must be taken into account to fully understand the cellular physiology in the co-culture [73]. The production and consumption of amino acids by both strains fulfilled the requirements for bidirectional amino acid exchange between the strains and allowed the manipulation of the co-culture by amino acid additions, such as methionine [67]. The amino acid consumption and production rates for *S. thermophilus* during co-cultivation with *L. bulgaricus* in the tube bioreactor system were compared with those of previously published data [62] for *S. thermophilus* during mono-culture growth (Figure 5). Basically, *S. thermophilus* released amino acids in co-culture to some extent (Figure 5), although these amino acids were available (Figure 3), indicating the uptake of peptides or amino acid synthesis (except glutamate, aspartate, and methionine). In contrast, *S. thermophilus* grown under mono-culture conditions only consumed amino acids (Figure 5). The dataset of this study confirmed the previously published simulated metabolic activities [67] of different *S. thermophilus* strains grown on various amino acid sources. The predicted amino acid fluxes were mostly within the same ranges as those presented in Figure 5. The measurements revealed the dynamics in the amino acid production and consumption of *S. thermophilus*, indicating the importance of extending the model when used for co-culture simulations [68,69,74].

Generally, the mutual release of almost all amino acids in an *L. bulgaricus*–*S. thermophilus* co-culture specified, for the first time, that both strains contribute to increasing amino acid concentrations in the medium and the enhanced current understanding of their metabolic activity. *L. bulgaricus* provided not only peptides but also—equally to *S. thermophilus*—amino acids to the co-culture, especially at the beginning of cultivation. At the end of the cultivation period, amino acid consumption occurred, indicating a switch between amino acid release and consumption.

Previous studies have revealed the upregulation of arginine biosynthesis genes in *S. thermophilus* [51,67,75], although arginine deficiency did not occur [67]. Consequently, here, it was hypothesised that arginine might serve as a precursor for ornithine or polyamine [67,75]. However, their low extracellular concentrations did not support the idea that arginine biosynthesis might have additional functions as a precursor [67]. The measurement of peptide-bound arginine in the compartment containing *S. thermophilus* revealed low arginine content (Figure 3). Thus, arginine upregulation may be caused by limiting arginine supply. In the compartment containing *S. thermophilus*, only 0.5% (after 8 h of continuous experiment) of all the analysed peptide-bound amino acids were arginine molecules (Figure 3). In contrast, the arginine fraction of casein represented 3% of the total casein-bound amino acids in a comparable experiment (Figure S12 in Supplementary Materials). This observation may indicate that either *L. bulgaricus* prefers the release of peptides from casein with low arginine content or that *S. thermophilus* favours the consumption of arginine-containing peptides. In either case, *S. thermophilus* likely faced arginine limitations during co-cultivation with *L. bulgaricus*. This observation supports the findings of previous studies [51,75] where an upregulation of arginine biosynthesis occurred in *S. thermophilus*.

Because 13-C glucose was used as a substrate in the medium, it was possible to distinguish between non-labelled amino acids hydrolysed from casein and 13-C amino acids synthesised from glucose. Measurements of the extracellular medium indicated that alanine, aspartate, tyrosine, and threonine were produced *de novo* from glucose. However, only the alanine pool was enriched with high amounts of 13-C alanine (Figure 6). A higher 13-C alanine concentration was measured in the *L. bulgaricus* compartment than in the compartment containing *S. thermophilus*. Metabolite balancing revealed that *L. bulgaricus* produced 13-C alanine, while *S. thermophilus* consumed 13-C alanine (Figure 6). This supported the hypothesis that *L. bulgaricus* might have an alanine transaminase [49] providing alanine to supply *S. thermophilus* or even serving as a signal molecule for *S. thermophilus* to indicate the presence of *L. bulgaricus*.

## 5. Conclusions

A new compartmentalised cultivation system was developed and established to unclose strain-specific metabolomics and the subsequent calculation of the production and consumption rates of strains grown in co-culture. This enabled the generation of experimental data for sophisticated models that allow comprehensive insight into cellular processes in co-cultures at a strain-specific level. Although the cultivation system was characterised by the spatial separation of cells, the adequate exchange of molecules, such as peptides and amino acids, was enabled. The experimental setting provided a sufficient volume for comprehensive sampling. The small size of the system reduced the preparation time and cost. However, only anaerobic cultivations were installed, to date. It is noteworthy that fairly similar growth characteristics were achieved in the compartmentalised approach compared to the one-pot co-cultivation approach.

The functionality of the system was demonstrated using an *S. thermophilus*–*L. bulgaricus* co-culture, indicating that both strains released and consumed amino acids. In addition, cultivation was performed using 13-C glucose to quantify amino acid production and consumption rates, as well as the *de novo* biosynthesis of amino acids, indicating alanine transaminase activity in *L. bulgaricus* and exchange with *S. thermophilus*.

This setup allowed the characterisation of interacting microorganisms and clarified the interaction fluxes between them, allowing the rational design of co-cultures. Using the compartmentalised system for the continuous cultivation of co-cultures opens the field for advanced co-culturing; for example, by applying technology for targeted evolution studies.

## Figures and Tables

**Table 1:** Comparison of mass balance terms for amino acids in the compartment containing *Streptococcus thermophilus*.

mean amino acid consumption	−*Q_i_*	3.0 ± 2.8 µM × h^−1^
mean amino acid dilution	*D* *× c_i_*	11.4 ± 10.1 µM × h^−1^
mean trans-membrane amino acid influx	*k_i_* × *g_i_*	5.5 ± 3.8 µM × h^−1^
mean change in amino acid concentration	*dc_i_*/*dt*	13.5 ± 13.6 µM × h^−1^
amino acid feed	*D* × *c_i,feed_*	0 µM × h^−1^ (feed medium without amino acids)
Damkoehler term for consumption	*Da_consumption_*	0.6 ± 0.4
Damkoehler term for dilution	*Da_dilution_*	2.3 ± 2.1
Damkoehler number	*Da_total_*	2.9 ± 2.3

*S. thermophilus* was co-cultivated with *Lactobacillus delbrueckii* subs. *bulgaricus* in the tube bioreactor system containing synthetic medium (SM) with glucose in the *S. thermophilus* compartment and SM with casein and glucose in the *L. bulgaricus* compartment.

## Data Availability

Not applicable.

## Supplementary Materials

The following supporting information can be downloaded at: https://www.mdpi.com/article/10.3390/bioengineering10010103/s1, Figure S1: Alanine concentrations measured to determine the transport coefficient; Figure S2: Amino acid transport coefficients *k*; Figure S3: Cultivation of *Streptococcus thermophilus* containing synthetic medium with casein and lactose in a crimp-top serum bottle; Figure S4: Growth of *Lactobacillus delbrueckii* subs. *Bulgaricus* (A) and *Streptococcus thermophilus* (B) in the vessel bioreactor system; Figure S5: Growth rate of *Lactobacillus delbrueckii* subs. *Bulgaricus* (A) and *Streptococcus thermophilus* (B) in the tube bioreactor system; Figure S6: Cell events of a co-culture grown in a crimp-top serum bottle; Figure S7: Biomass and growth rate of *Streptococcus thermophilus* in the tube bioreactor system; Figure S8: Glucose and lactate concentrations in the compartment containing *Streptococcus thermophilus*; Figure S9: Fractions of alanine isotopologues; Figure S10: Diffusion rate of 13-C alanine across the membrane in the tube bioreactor system; Figure S11: *r*-values at the outlet of the membrane unit; Figure S12: Amino acid composition of casein; Table S1: Composition of the synthetic medium; Table S2: Amino acid concentrations to determine the transport coefficient (*k*); Table S3: Amino acid concentrations in the *Streptococcus thermophilus* compartment and the *Lactobacillus delbrueckii* subs. *bulgaricus* compartment cultivated in the tube bioreactor system; Code S1: Determination of the amino acid transport coefficient by the least-square estimate in MATLAB ^®^.
