# Supplementary material for: A Two-Compartment Fermentation System to Quantify Strain-Specific Interactions in Microbial Co-Cultures
Source: Bioengineering (Basel). 2023 Jan 11;10(1):103. doi: 10.3390/bioengineering10010103 (PMC9854596; doi:10.3390/bioengineering10010103)
Supplement: Supplementary file 1 [file bioengineering-10-00103-s001.zip › bioengineering-2092713-supplementary.pdf]

bioengineering

MDPI

Article

A Two-Compartment Fermentation System to Quantify Strain-Specific Interactions in Microbial Co-Cultures

Andreas Ulmer [1], Stefan Veit [1], Florian Erdemann [1], Andreas Freund [1], Maren Loesch [1], Attila Teleki [1], Ahmad A. Zeidan [2] and Ralf Takors [1,*]

[1] Institute of Biochemical Engineering, University of Stuttgart, 70569 Stuttgart, Germany
[2] Systems Biology, R&D Discovery, Chr. Hansen A/S, 2970 Hørsholm, Denmark
* Correspondence: takors@ibvt.uni-stuttgart.de

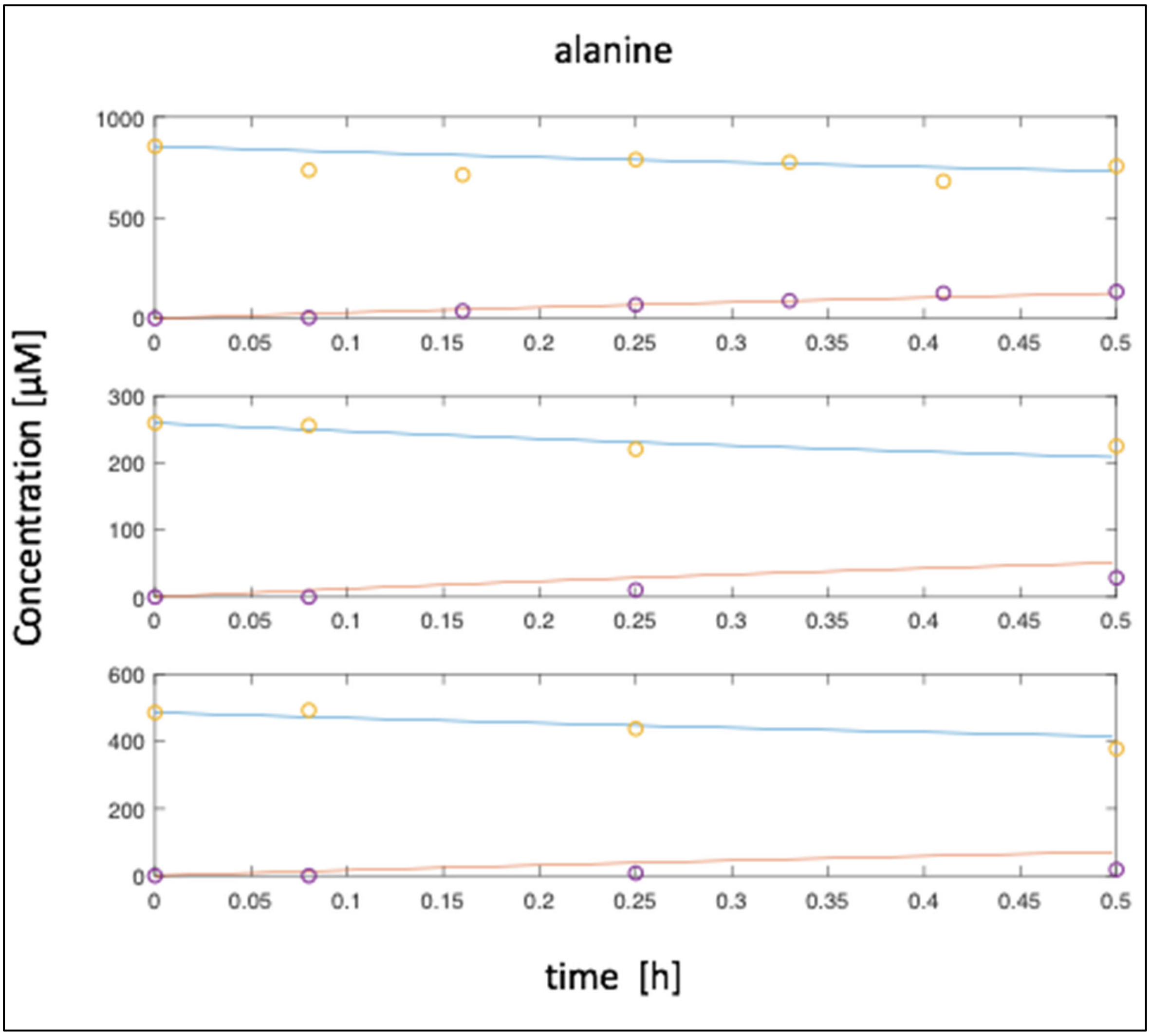

Figure S1. Alanine concentrations are measured to determine the transport coefficient $k_{alanine}$ (purple circles: amino acid concentrations in compartment 1; orange circles: amino acid concentrations compartment 2). The process model was implemented in Matlab® and $k_{alanine}$ was optimized. Lines: Simulated alanine concentrations based on initial alanine concentration at t = 0 hours and estimated $k_{alanine}$ = 0.34 h^{-1} for three independent experiments with different initial alanine concentrations.

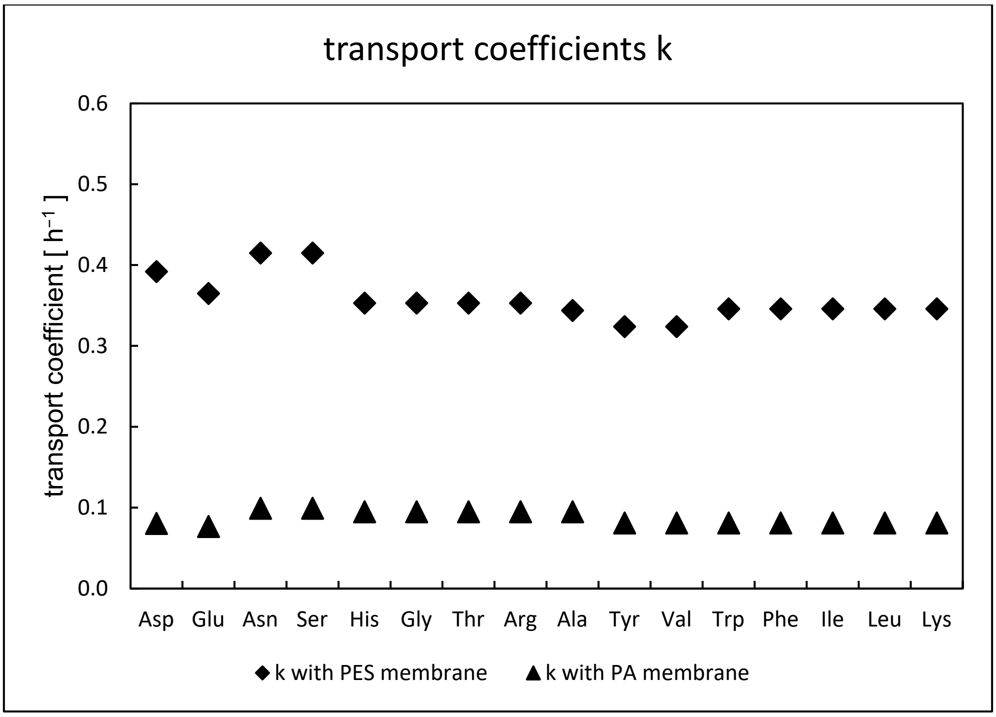

Figure S2. Amino acid transport coefficients k_i estimated for the membrane unit with an integrated PES (**rhomb**) or PA (**triangle**) membrane, attached to the vessel bioreactor system.

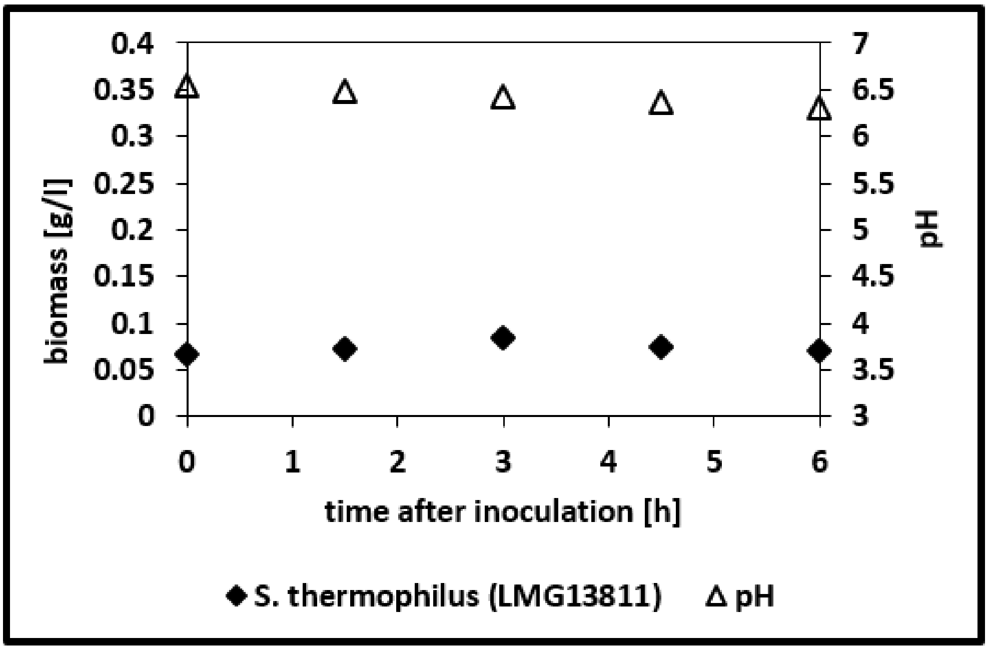

Figure S3. Cultivation of *S. thermophilus* in a crimp-top serum bottle containing synthetic medium with casein and lactose. Biomass was monitored by flow cytometry (rhomb).

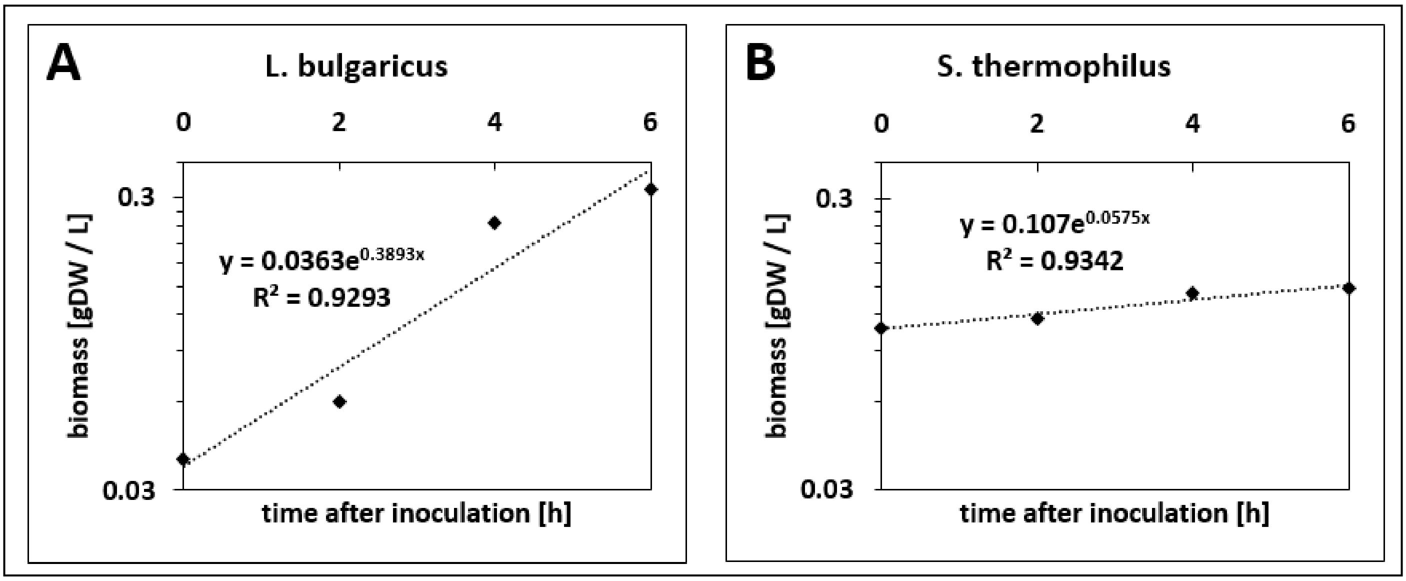

Figure S4. Growth of *L. bulgaricus* (**A**) and *S. thermophilus* (**B**) in vessel bioreactor system. The *S. thermophilus*-compartment was filled with SM + lactose, and the *L. bulgaricus*-compartment was filled with SMcas + lactose. Samples were analysed by flow cytometry to calculate biomass. Growth rates were calculated from zero to six hours: μ (*S. thermophilus*) = 0.06 h^{-1} and μ (*L. bulgaricus*) = 0.39 h^{-1}.

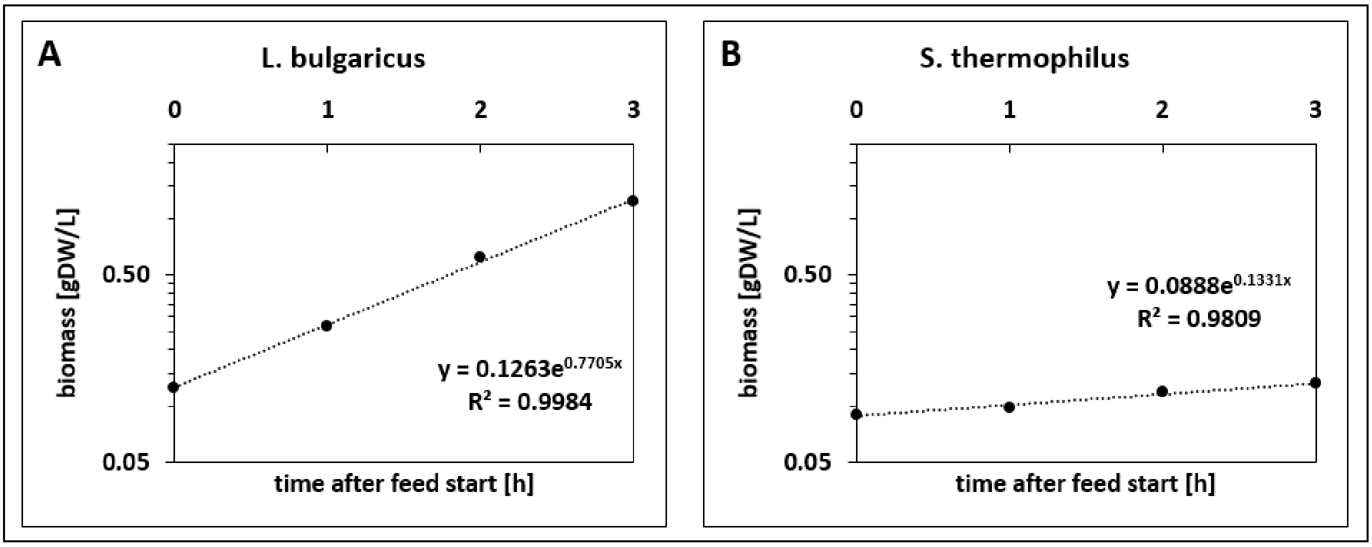

Figure S5. Growth rate of *L. bulgaricus* (**A**) and *S. thermophilus* (**B**) in tube-bioreactor-system. The *S. thermophilus*-compartment was filled with SM + lactose, and the *L. bulgaricus*-compartment was filled with SMcas + lactose. Samples were analysed by flow cytometry to calculate biomass. The dilution rate for each compartment was D = 0.14 h^{-1}. This results in μ(*L. bulgaricus*) = 0.77 h^{-1} + 0.14 h^{-1} = 0.91 h^{-1}, and μ(*S. thermophilus*) = 0.13 h^{-1} + 0.14 h^{-1} = 0.27 h^{-1}.

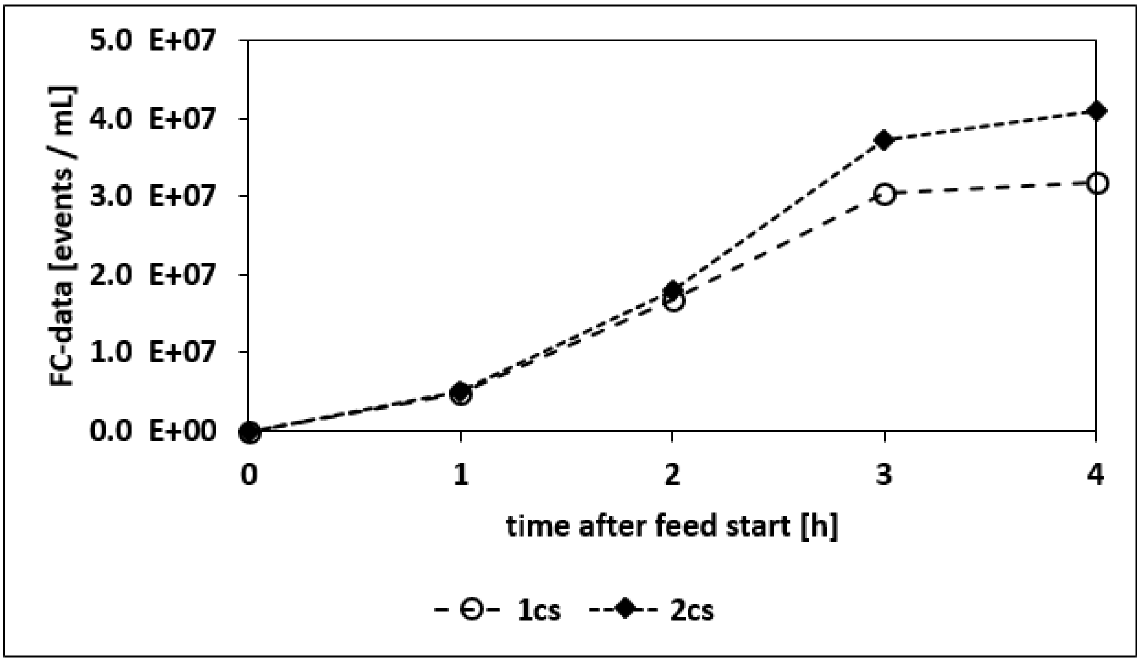

Figure S6. (**open circles**) Cell events of a co-culture grown in a crimp-top serum bottle measured by flow cytometry. (**filled rhomb**) The biomass in each compartment of a tube-bioreactor-system was analysed by flow cytometry and summed up. The initial FC-data (t = 0 hour) were subtracted from all values to normalize and compare data sets.

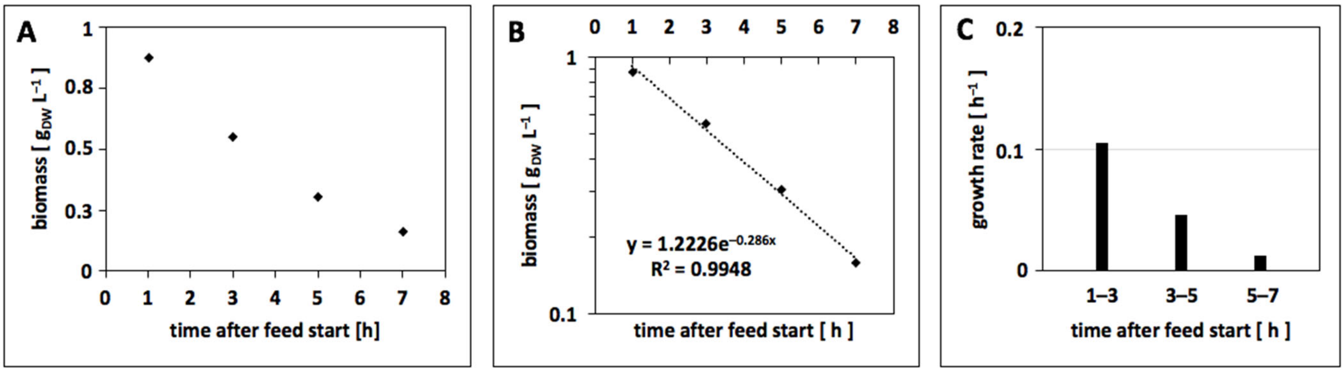

Figure S7. (**A**) Biomass of *S. thermophilus* in tube-bioreactor-system. The *S. thermophilus*-compartment was filled with SM + glucose, and the *L. bulgaricus*-compartment was filled with SMcas+glucose. Biomass in *S. thermophilus*-compartment was monitored by optical density. (**B**) Calculated growth rate μ with a dilution rate of D = 0.34 h^{-1} results in μ = (- 0.29 h^{-1} + 0.34 h^{-1}) = 0.05 h^{-1}. (**C**) Calculated growth rate μ for each time step.

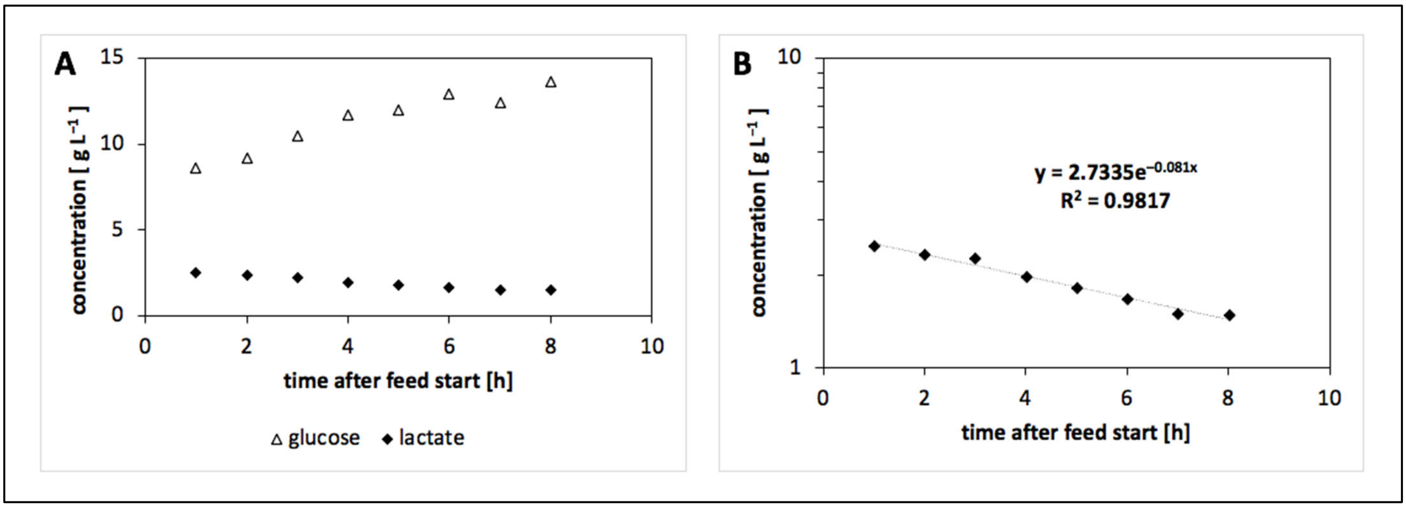

Figure S8. Glucose and lactate concentrations in *S. thermophilus*-compartment measured by HPLC. (**A**) Strains were cultivated in tube-bioreactor-system filled with SMcas+glucose in the *L. bulgaricus*-compartment ($D = 0.07\ h^{-1}$) and SM+glucose in the *S. thermophilus*-compartment ($D = 0.34\ h^{-1}$). (**B**) Lactate production rate in *S. thermophilus*-compartment (considering $D = 0.34\ h^{-1}$): $r = -0.08\ h^{-1} + 0.34\ h^{-1} = 0.26\ h^{-1}$.

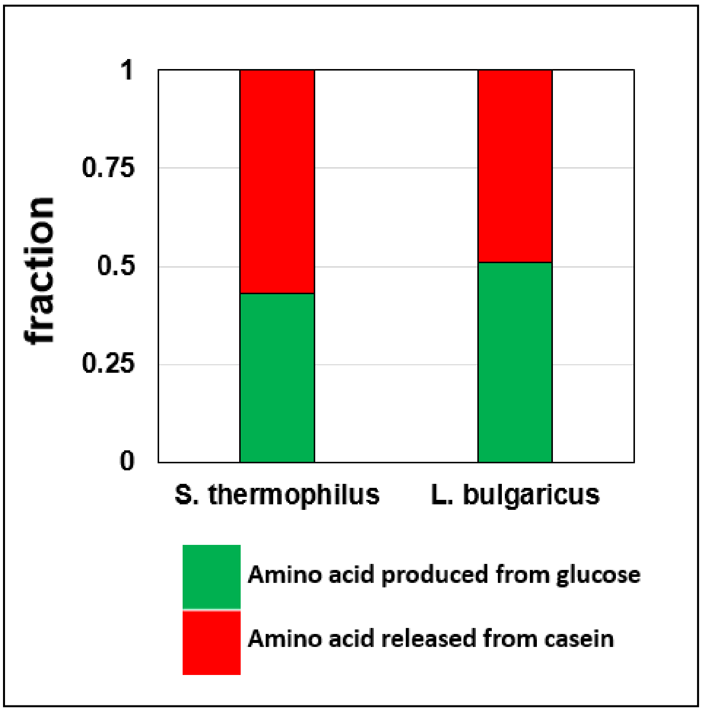

Figure S9. Fractions [mol/mol] of alanine isotopologues: m + 0, m + 1, and m + 2 were corrected for natural isotopologues. Fully 13-C labeled (m + 3) alanine is shown in red. The green fraction indicates alanine hydrolysed from casein and the red fraction indicates alanine produced from glucose. Strains were cultivated in tube-bioreactor-system containing SMcas + 13-C glucose in the *L. bulgaricus*-compartment, and SM + 13-C glucose in the *S. thermophilus*-compartment. After 24 hours, intracellular metabolites were analysed from strains in each compartment by LC-MS/MS.

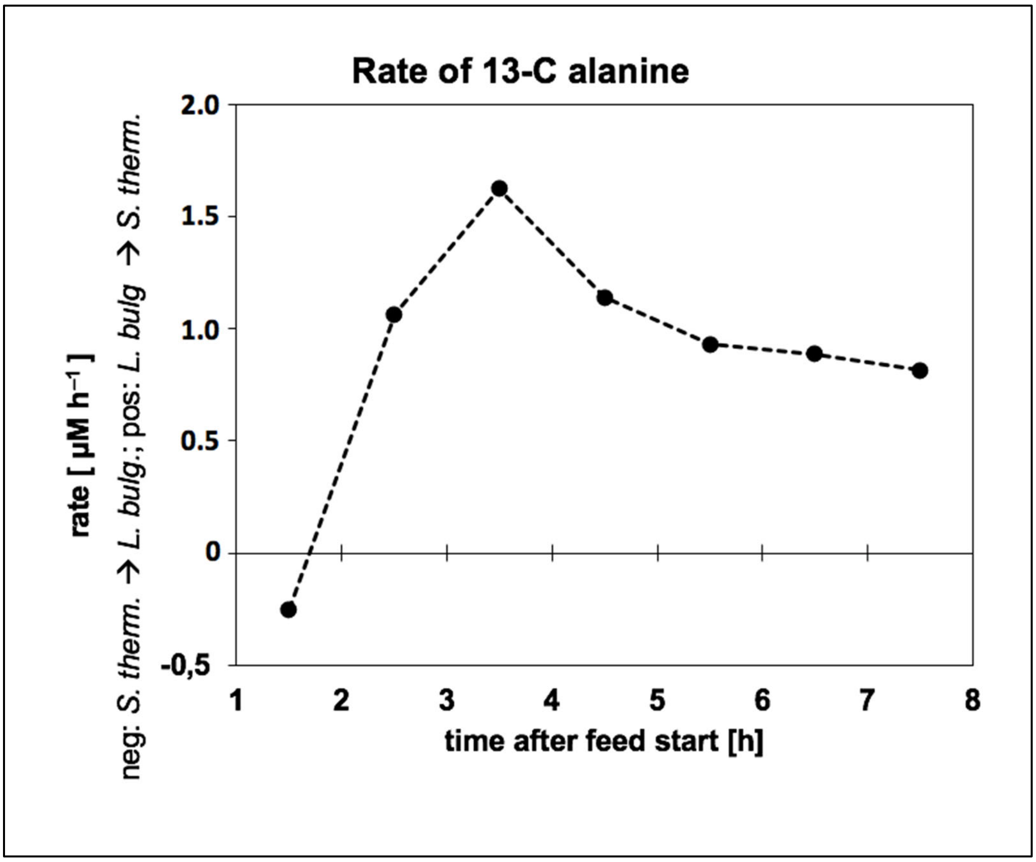

Figure S10. Diffusion rate of 13-C alanine across the membrane in tube-bioreactor-system filled with *L. bulgaricus* and *S. thermophilus*. Negative rates: diffusion from *S. therm*ophilus-compartment to *L. bulgaricus*-compartment. Positive rates: diffusion from *L. bulgaricus*-compartment to *S. therm*ophilus-compartment.

A

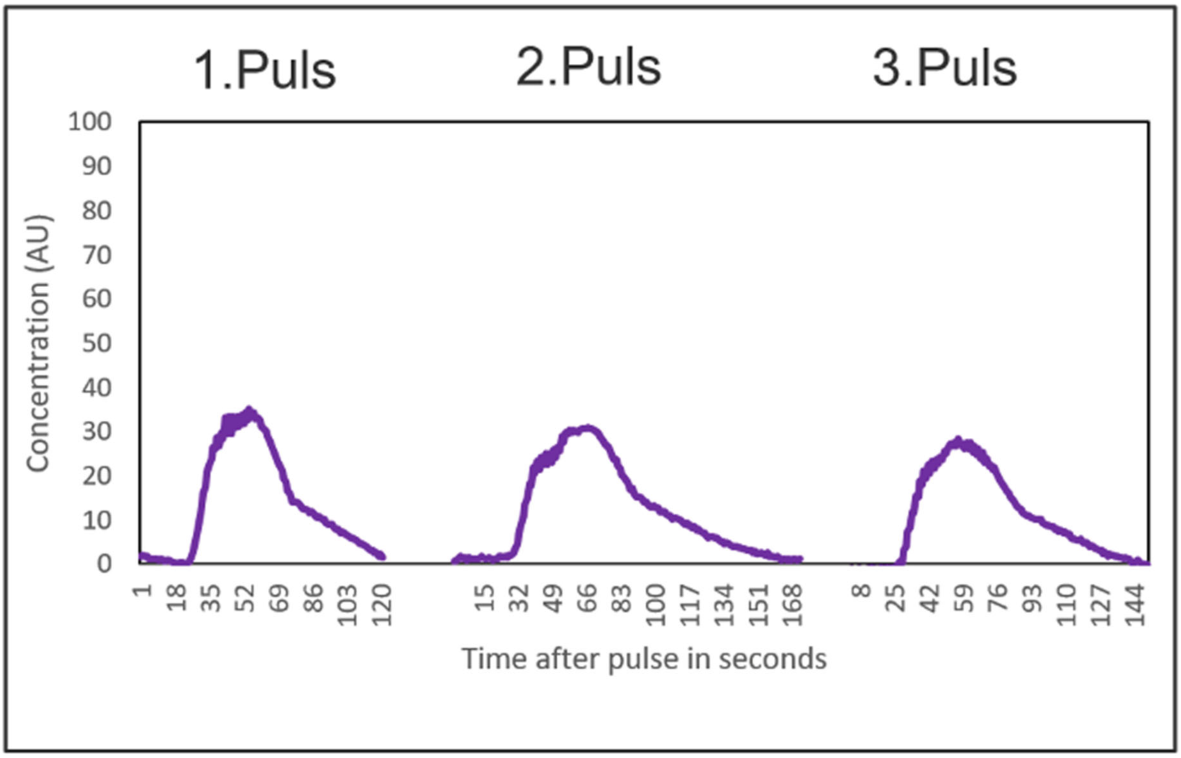

B

	$Area_{OUT}$	$Bodenstein_{OUT}$	τ [s]	σ [s]	σ^2 [s^2]
**1. Puls**	1637	20	68	22	477
**2. Puls**	1636	16	87	31	975
**3. Puls**	1562	19	78	25	643
**Mean**	**1612 ± 43**	**18 ± 2**	**77 ± 10**	**26 ± 5**	**698 ± 254**

Figure S11. (**A**) *r*-values at the outlet of the membrane unit (violet curve). Change of the *r*-values was induced by a hydrochloric acid pulse at zero second. Three pulses were recorded to calculate the Bodenstein number in the membrane unit. (**B**) Calculated numbers to determine the Bodenstein number.

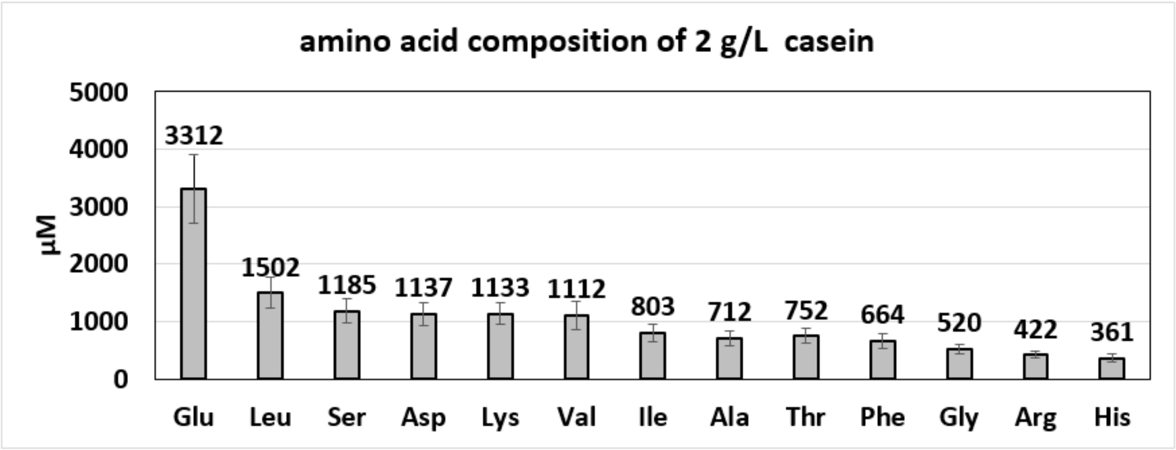

Figure S12. Amino acid composition of casein. 2 g/L casein was hydrolysed with HCL and amino acid concentrations were measured by HPLC. Numbers on top of each bar indicate exact concentration.

Table S1. Composition of the synthetic medium (SM). The SM contains all listed compounds, except amino acids or casein, and glucose or lactose, respectively.

Category	Compound	Concentration [g L^{-1}]	CAS Number
-	Di-potassium hydrogen phosphate	2.5	7758-11-4
	Potassium dihydrogen phosphate	3	7778-77-0
	Sodium acetate	1	127-09-3
	Ammonium citrate tribasic	0.6	3458-72-8
	Manganese sulfate monohydrate	0.02	10034-96-5
	Iron(II) sulfate heptahydrate	0.00132	7782-63-0
	Calcium chloride dihydrate	0.08745	10035-04-8
	Tween 80	1 mL/L	9005-65-6
	D-Lactose monohydrate	15.75	10039-26-6
	Glucose monohydrate	15.75	14431-43-7
	Magnesium sulfate heptahydrate	0.2	10034-99-8
	Urea	0.12	57-13-6
nucleobases	Adenine	0.01	73-24-5
	Guanine	0.01	73-40-5
	Uracil	0.01	66-22-8
	Xanthine	0.01	69-89-6
vitamins	Biotin	0.0002	58-85-5
	Folic acid	0.0002	59-30-3
	Pyridoxal hydrochloride	0.001	65-22-5
	Riboflavin	0.0005	83-88-5
	Thiamine chloride hydrochloride	0.0005	67-03-8
	Nicotinamide	0.0005	98-92-0
	Cyanocobalamin	0.0005	68-19-9
	4-Aminobenzoic acid	0.0005	150-13-0
	D-Pantothenic acid hemicalcium salt	0.004	137-08-6
	DL-6,8-thioctic acid	0.0005	1077-28-7
trace elements	Ammonium molybdate tetrahydrate	0.0000037	12054-85-2
	Cobalt(II) chloride hexahydrate	0.000007	7791-13-1
	Boric acid	0.000025	10043-35-3
	Copper(II) sulfate pentahydrate	0.0000025	7758-99-8
	Zinc sulfate heptahydrate	0.0000029	7446-20-0
amino acids	L-Alanine	0.1	56-41-7
	L-Arginine	0.317	74-79-3
	L-Asparagine monohydrate	0.343	5794-13-8
	L-Aspartic acid	0.499	56-84-8
	L-Cysteine hydrochloride monohydrate	0.3	7048-04-6
	L-Glutamic acid	0.331	56-86-0
	L-Glutamine	0.29	56-85-9
	Glycine	0.16	56-40-6
	L-Histidine monohydrochloride monohydrate	0.273	5934-29-2
	L-Isoleucine	0.361	73-32-5

	L-Leucine	0.6	61-90-5
	L-Lysine	0.351	56-87-1
	L-Methionine	0.119	63-68-3
	L-Phenylalanine	0.34	63-91-2
	L-Proline	0.921	147-85-3
	L-Serine	0.359	56-45-1
	L-Threonine	0.3	72-19-5
	L-Tryptophan	0.102	73-22-3
	L-Tyrosine	0.12	60-18-4
	L-Valine	0.468	72-18-4
casein	Casein	2	9005-46-3

Table S2. Amino acid concentrations (μM) to determine transport coefficient k.

	t [h]	Asp	Glu	Asn	Ser	His	Gly	Thr	Arg	Ala	Tyr	Val	Trp	Phe	Ile	Leu	Lys
**PES Expt.1 comp1**	0	3018	1788	1086	2862	884	2372	1816	1302	855	461	2508	337	1323	1874	3372	1644
	0	2636	1554	953	2459	771	1911	1581	1122	736	400	2149	293	1151	1641	2913	1421
	0	2548	1513	946	2343	762	1831	1528	1111	713	387	2134	297	1155	1615	2843	1425
	0	2820	1671	1023	2619	828	2052	1695	1218	789	434	2334	322	1245	1763	3127	1565
	0	2779	1646	1010	2575	817	2001	1671	1202	776	427	2313	318	1240	1749	3097	1551
	0	2424	1446	902	2254	728	1791	1461	1068	681	376	2007	281	1100	1532	2717	1360
	1	2732	1619	1002	2498	807	1910	1634	1186	757	417	2265	317	1227	1714	3025	1539
**PES Expt. 1 comp2**	0	0	0	0	0	0	0	0	0	0	0	0	0	0	0	0	0
	0	24	12	4	21	2	14	12	5	4	0	22	0	8	13	24	5
	0	153	83	57	121	39	76	85	52	37	17	128	14	66	90	147	66
	0	271	149	101	213	71	131	152	96	67	34	229	27	117	161	261	124
	0	346	193	134	274	95	172	195	127	87	46	292	35	155	210	340	163
	0	496	276	190	388	135	239	279	182	125	67	420	52	219	299	481	236
	1	517	288	194	420	142	265	295	192	133	70	437	54	224	311	507	246
**PES Expt. 2 comp1**	0	1000	579	387	803	294	505	579	416	259	148	887	115	460	632	1040	530
	0	1003	573	376	795	287	485	575	407	256	147	871	115	453	623	1020	530
	0	856	495	330	686	252	434	493	357	220	127	755	99	398	541	893	457
	1	880	506	339	701	259	439	506	369	225	131	776	104	413	555	913	478
**PES Expt.2 comp2**	0	0	0	0	0	0	0	0	0	0	0	0	0	0	0	0	0

	0	4	1	0	5	0	3	0	0	0	0	6	0	1	2	6	0
	0	52	28	18	42	13	26	28	18	11	2	47	3	23	32	54	22
	1	122	67	44	97	33	59	67	44	29	14	105	11	53	73	121	57
**PES Expt.3 comp1**	0	1805	1063	688	1548	541	1076	1063	778	486	279	1523	214	834	1143	1938	999
	0	1874	1087	701	1557	546	1034	1084	785	493	284	1564	217	842	1164	1945	1003
	0	1648	961	626	1365	488	915	959	699	438	248	1399	192	752	1048	1748	896
	1	1458	835	545	1174	421	741	846	599	379	216	1277	169	657	914	1498	776
**PES Expt.3 comp2**	0	0	0	0	0	0	0	0	0	0	0	0	0	0	0	0	0
	0	6	2	0	6	0	3	1	0	0	0	6	0	1	2	6	0
	0	40	20	13	33	9	22	21	10	8	2	35	0	15	22	38	14
	1	79	40	28	65	18	43	43	23	18	7	65	5	32	44	74	31
**PA Expt.1 comp1**	0	1089	628	419	877	320	555	628	456	280	162	952	131	510	685	1129	598
	0	1030	590	385	842	296	541	593	426	266	150	877	119	468	639	1072	555
	0	1238	724	386	1219	302	779	723	522	329	188	1146	147	566	778	1306	546
	1	1141	666	366	1095	282	679	666	484	299	175	1111	138	534	724	1195	507
**PA Expt. 1 comp2**	0	2	1	0	1	0	1	0	0	0	0	11	3	2	1	2	0
	0	12	7	4	11	2	8	7	4	3	2	20	1	7	8	12	4
	0	36	20	12	35	8	23	21	12	10	5	41	4	17	22	35	13
	1	72	39	23	69	16	46	41	24	19	10	73	7	32	42	68	26
**PA Expt. 2 comp1**	0	2043	1226	652	2190	520	1556	1236	910	573	317	1913	248	963	1313	2293	964

	0	1933	1156	618	2047	490	1425	1163	859	535	299	1784	238	915	1233	2146	921
	0	1807	1075	567	1902	450	1303	1082	788	497	277	1658	217	837	1145	1989	842
	1	1635	968	521	1698	411	1149	977	718	446	252	1512	203	777	1042	1792	775
**PA Expt.2 comp2**	0	3	1	0	2	0	1	1	1	1	0	12	3	2	2	3	1
	0	8	4	3	8	2	6	4	0	3	1	17	3	1	5	8	3
	0	14	7	4	13	3	10	7	4	4	2	20	1	6	8	12	3
	1	19	10	6	19	4	15	10	5	5	3	25	2	8	10	16	5
**PA Expt.3 comp1**	0	3132	1918	985	3714	805	3117	1935	1454	914	471	2896	371	1454	1991	3680	1536
	0	2917	1779	920	3368	746	2699	1789	1343	841	444	2664	349	1362	1855	3384	1425
	0	2697	1636	843	3085	684	2439	1644	1228	773	407	2473	318	1247	1707	3107	1300
	1	2668	1616	828	3030	671	2344	1623	1203	757	415	2445	313	1227	1683	3073	1281
**PA Expt.3 comp2**	0	8	2	1	3	0	1	2	1	1	1	12	3	2	3	3	1
	0	26	14	9	26	7	18	15	10	7	4	34	3	13	17	26	11
	0	69	38	25	75	18	53	43	30	21	10	78	7	34	46	74	32
	1	138	77	50	151	36	105	87	59	42	20	146	18	66	91	147	62
	0	2768	1962	922	3088	73	2411	1663	1264	780	435	2531	335	1322	1781	3182	1300
	2	2512	1762	818	2682	67	1903	1508	1142	681	408	2335	314	1231	1643	2881	1165
	4	2408	1678	720	2481	65	1684	1411	1080	602	381	2173	296	1148	1534	2697	1115
	0	7	3	1	4	0	4	2	4	4	1	17	2	2	3	4	2
	2	613	293	4	660	8	427	406	238	82	83	587	74	300	378	657	227
	4	1011	483	47	1049	9	673	688	392	107	137	953	122	499	613	1074	368

Table S3. Amino acid concentrations (µM) in *S. thermophilus*-compartment and *L. bulgaricus*-compartment cultivated in tube-bioreactor-system analysed by LC-MS/MS.

**sample**	**time after feed start**	glycine	alanine	serine	proline	valine	leucine	isoleucine	tyrosine	asparate	threonine	lysine	glutamate	methionine	phenylalanine	arginine	tryptophan
µM	hour	µM	µM	µM	µM	µM	µM	µM	µM	µM	µM	µM	µM	µM	µM	µM	µM
S. therm. compartment	1	68	9	124	708	206	386	115	59	34	152	124	40	10	109	140	21
S. therm. compartment	2	57	9	101	677	188	366	100	53	24	137	110	27	5	98	130	18
S. therm. compartment	3	49	6	75	613	153	321	80	43	19	102	86	21	1	79	113	13
S. therm. compartment	4	38	4	57	559	125	281	62	37	13	74	62	19	0	65	96	10
S. therm. compartment	5	40	4	49	522	106	249	50	33	11	56	54	17	0	54	87	8
S. therm. compartment	6	26	3	39	482	80	197	34	27	8	38	40	14	0	39	72	6
S. therm. compartment	7	30	3	34	439	66	165	25	25	7	25	31	12	0	32	64	5
S. therm. compartment	8	31	4	29	388	52	129	18	21	6	18	26	9	0	22	56	4
L. bulg. compartment	1	26	4	39	358	66	166	33	18	11	36	48	17	4	30	73	5
L. bulg. compartment	2	42	11	81	562	142	299	77	42	32	82	89	49	1	69	116	13

L. bulg. compartment	3	58	17	111	675	192	368	107	64	34	113	114	63	0	96	131	18
L. bulg. compartment	4	62	12	91	639	169	340	91	56	24	94	100	45	0	85	121	15
L. bulg. compartment	5	48	9	71	587	138	297	70	47	17	68	78	31	0	67	104	12
L. bulg. compartment	6	52	9	60	551	115	261	54	41	13	47	61	24	0	52	94	10
L. bulg. compartment	7	46	8	49	492	87	209	36	33	10	27	43	17	0	34	77	7
L. bulg. compartment	8	39	8	41	458	75	173	26	30	8	16	32	15	0	24	68	7

Code S1: Determination of amino acid transport coefficient by least-square estimate in Matlab®.

```
% this code was written to determine the transport koefficient ki based on amino acid experiements
clear
error_best = 10000000;
load('data_for_k_PES.mat'); % amino acid concentrations
% Column Amino Acid
% Asp Glu Asn     Ser     His     Gly     Thr     Arg     Ala     Tyr     Val     Trp     Phe     Ile     Leu     Lys
for col = 1:1:16;
data_for_k_one_aa = data_for_k(:,col);
for i = 0.2:0.001:0.5 % change ki
parameters = i;
% Time
time_delta = 0.083/10;
time_span    = 0:time_delta:0.5; % in hours
%% FIRST EXPERIMENT
% initial conditions
c_aa_comp_1 = data_for_k_one_aa(1,1); % first expt
c_aa_comp_2 = 0;
c = [c_aa_comp_1 c_aa_comp_2];
% Integration
options = odeset('NonNegative',[1 2]);
f=@(t,x)balanceEquation(x,parameters);
[T1,Conc1] = ode45(f,time_span,c,options);
%% Second EXPERIMENT
% initial conditions
c_aa_comp_1 = data_for_k_one_aa(15,1); % first expt
c_aa_comp_2 = 0;
c = [c_aa_comp_1 c_aa_comp_2];
% Integration
options = odeset('NonNegative',[1 2]);
f=@(t,x)balanceEquation(x,parameters);
[T2,Conc2] = ode45(f,time_span,c,options);
%% Thirds EXPERIMENT
% initial conditions
c_aa_comp_1 = data_for_k_one_aa(23,1); % first expt
c_aa_comp_2 = 0;
c = [c_aa_comp_1 c_aa_comp_2];
% Integration
options = odeset('NonNegative',[1 2]);
f=@(t,x)balanceEquation(x,parameters);
[T3,Conc3] = ode45(f,time_span,c,options);
```

```
%% ERROR
error = abs(Conc1(11,2)- data_for_k_one_aa(9,1))+...
    abs(Conc1(21,2)- data_for_k_one_aa(10,1))+...
    abs(Conc1(31,2)- data_for_k_one_aa(11,1))+...
    abs(Conc1(41,2)- data_for_k_one_aa(12,1))+...
    abs(Conc1(51,2)- data_for_k_one_aa(13,1))+...
        abs(Conc1(61,2)- data_for_k_one_aa(14,1))+...
    abs(Conc2(11,2)- data_for_k_one_aa(20,1))+...
    abs(Conc2(31,2)- data_for_k_one_aa(21,1))+...
      abs(Conc2(61,2)- data_for_k_one_aa(22,1))+...
    abs(Conc3(11,2)- data_for_k_one_aa(28,1))+...
    abs(Conc3(31,2)- data_for_k_one_aa(29,1))+...
    abs(Conc3(61,2)- data_for_k_one_aa(30,1));
if error <= error_best
    error_best = error;
    k = parameters;
end
end
%% PLOT
% Integration
figure
subplot(3,1,1)
parameters = k ;
% initial conditions
c_aa_comp_1 = data_for_k_one_aa(1,1); % first expt
c_aa_comp_2 = 0;
c = [c_aa_comp_1 c_aa_comp_2];
% Integration
options = odeset('NonNegative',[1 2]);
f=@(t,x)balanceEquation(x,parameters);
[T1,Conc1] = ode45(f,time_span,c,options);
t_meas = [0 0.08 0.16 0.25 0.33 0.41 0.5];
c_meas_1_2 = data_for_k_one_aa(1:7,1);
c_meas_2_2 = data_for_k_one_aa(8:14,1);
plot(T1,Conc1)
hold on
scatter(t_meas,c_meas_1_2)
hold on
scatter(t_meas,c_meas_2_2)
% SEC EXPT
subplot(3,1,2)
parameters = k ;
```

```
% initial conditions
c_aa_comp_1 = data_for_k_one_aa(15,1); % first expt
c_aa_comp_2 = 0;
c = [c_aa_comp_1 c_aa_comp_2];
% Integration
options = odeset('NonNegative',[1 2]);
f=@(t,x)balanceEquation(x,parameters);
[T1,Conc1] = ode45(f,time_span,c,options);
t_meas = [0 0.08    0.25    0.5];
c_meas_1_3 = data_for_k_one_aa(15:18,1);
c_meas_2_3 = data_for_k_one_aa(19:22,1);
plot(T1,Conc2)
hold on
scatter(t_meas,c_meas_1_3)
hold on
scatter(t_meas,c_meas_2_3)
% THIRD EXPT
subplot(3,1,3)
parameters = k ;
% initial conditions
c_aa_comp_1 = data_for_k_one_aa(23,1); % first expt
c_aa_comp_2 = 0;
c = [c_aa_comp_1 c_aa_comp_2];
% Integration
options = odeset('NonNegative',[1 2]);
f=@(t,x)balanceEquation(x,parameters);
[T1,Conc3] = ode45(f,time_span,c,options);
t_meas = [0 0.08    0.25    0.5];
c_meas_1 = data_for_k_one_aa(23:26,1);
c_meas_2 = data_for_k_one_aa(27:30,1);
plot(T1,Conc3)
hold on
scatter(t_meas,c_meas_1)
hold on
scatter(t_meas,c_meas_2)
%%
k_total(col) = k;
end
%%
function dc_dt = balanceEquation(x0,parameters)
k = parameters(1);
dc_dt = zeros(2,1);
```

```
c_aa_comp_1 = x0(1);
c_aa_comp_2 = x0(2);
dc_dt(1) = k * (c_aa_comp_2 - c_aa_comp_1);
dc_dt(2) = k * (c_aa_comp_1 - c_aa_comp_2);
end
```
